# Prediction of novel target genes and pathways involved in tall cell variant papillary thyroid carcinoma

**DOI:** 10.1097/MD.0000000000013802

**Published:** 2018-12-21

**Authors:** Fada Xia, Bo Jiang, Yong Chen, Xin Du, Yao Peng, Wenlong Wang, Zhuolu Wang, Xinying Li

**Affiliations:** Department of General surgery, Xiangya Hospital, Central South University, Changsha, China.

**Keywords:** hub gene, papillary thyroid carcinoma, protein-protein interaction network, ROC, tall cell variant

## Abstract

**Background:**

Tall cell variant papillary thyroid carcinoma (TCPTC) is reportedly associated with aggressive clinicopathological parameters and poor outcomes; however, the molecular mechanisms underlying TCPTC remain poorly understood.

**Methods:**

The gene mutation types and mRNA expression profiles of patients with TCPTC were obtained from The Cancer Genome Atlas (TCGA) database. Differentially expressed genes (DEGs) were identified. Pathways in the interaction network and the diagnostic approaches of candidate markers for TCPTC were investigated.

**Results:**

*BRAF* mutation was particularly prevalent in TCPTC with a mutation frequency of 78%. TCPTC was associated with a patient age >45 years, tumor multifocality, extrathyroidal extension, a higher T stage, advanced AJCC TNM stages, *BRAF* V600E mutation, and poor disease-free survival. We identified 4138 TCPTC-related DEGs and 301 TCPTC-specific DEGs. Intriguingly, the gene expression pattern revealed that the dysregulated levels of both putative oncogenes and tumor suppressors in TCPTC were higher than those in classical/conventional variant PTC (cPTC). Functional enrichment analyses revealed that these DEGs were involved in several cancer-related pathways. A protein-protein interaction (PPI) network was constructed from the 301 TCPTC-specific DEGs, and 3 subnetworks, and 8 hub genes were verified. Receiver operating characteristic (ROC) analyses revealed that 6 hub genes, including *COL5A1*, *COL1A1*, *COL10A1*, *COL11A1*, *CCL20*, and *CXCL5*, could be used not only for the differential diagnosis of PTC from normal samples, but also for the differential diagnosis of TCPTC from cPTC samples.

**Conclusions:**

Our study might provide further insights into the investigations of the tumorigenesis mechanism of TCPTC and assists in the discovery of novel candidate diagnostic markers for TCPTC.

## Abstract 2

Supplemental Digital Content is available in the text

## 1 Introduction

Papillary thyroid carcinoma (PTC) is the most common endocrine neoplasm and the most frequent malignant thyroid tumor. PTC consists of several histological variants, the most common of which are classical/conventional variant PTC (cPTC), follicular- variant PTC (FVPTC), and tall cell variant PTC (TCPTC), which collectively account for the vast majority of PTCs.^[1,2]^ Some rare PTC variants, such as Hobnail, Columnar, Solid and diffuse sclerosing variant PTC, have also been reported.^[3,4]^ Most patients with PTC have excellent prognoses, but some histological variants of PTC, such as TCPTC, are characterized by more aggressive phenotypes.^[2,5]^ TCPTC consists predominantly of tall cells (TC), which have a height of more than at least double their width, as well as an eosinophilic cytoplasm and hyperchromatic basilar nuclei.^[1,6]^ The incidence of TCPTC ranges from 2% to 19% among PTC patients described in the literatures.^[5,7,8]^

The clinical characteristics and prognosis of TCPTC have been reported by a number of studies. The consensus is that TCPTC is related to aggressive clinicopathological parameters, including tumor stage, tumor multifocality, extrathyroidal extension, lymphovascular invasion, initial lymph node metastasis, pre-ablation lung metastasis, and *BRAF* mutations.^[1,5,6,9–11]^ However, studies on the outcomes of patients with TCPTC have revealed controversial results.^[5,7,11,12]^ Although numerous studies that have investigated the biological characteristics of TCPTC,^[13,14]^ the molecular mechanisms underlying TCPTC remain poorly understood. Therefore, recognizing molecular alterations and identifying novel diagnostic biomarkers for TCPTC is important. A comprehensive and systematic analysis of gene expression profiles in TCPTC is also lacking. The Cancer Genome Atlas (TCGA) database catalogs genetic information, including microRNA and mRNA expression profiles, somatic mutations and copy number variations, and covers 33 types of cancers, thereby facilitating studies on gene expression signatures and tumorigenesis mechanisms.^[15]^

In this study, the gene mutation and mRNA expression profiles of patients with TCPTC from TCGA database were obtained by RNA sequencing. The clinical features and prognoses of TCPTC patients were examined. Differentially expressed genes (DEGs) were identified, and functional enrichment and pathway analyses were performed. Subsequently, protein-protein interaction (PPI) networks were constructed to identify hub genes. We also performed a receiver operating characteristic (ROC) analysis to evaluate the diagnostic values of the candidate genes. Our study may provide a meaningful contribution towards exploring the tumorigenesis mechanism of TCPTC and assisting in the discovery of novel candidate diagnostic markers for TCPTC.

## 2 Materials and methods

### 2.1 TCGA data access

The standardized level 3 RNA sequencing data of PTC patients and the corresponding clinical records in TCGA were obtained from the cBioPortal for Cancer Genomics (http://www.cbioportal.org/index.do) and FireBrowse (http://firebrowse.org). Among the total 507 cases in TCGA thyroid carcinoma (TCGA-THCA) dataset, there were 359 cPTCs, 37 TCPTCs (≥50% tall cell feature) and 59 normal tissue samples. A total of 357 cPTC and 35 TCPTC samples with complete mRNA sequencing and clinical data and 59 normal tissue samples were included in the subsequent analyses (the case IDs are shown in Supplementary Table S1). The RNAseq by Expectation-Maximization (RSEM) values were utilized to quantify the mRNA expression levels. Histological images of all samples are openly available on the Cancer Slide Digital Archive website (CSDA, http://cancer.digitalslidearchive.net).

### 2.2 Identification of DEGs

Differentially expressed mRNAs between the PTC (including cPTC and TCPTC) and normal tissues, and between the TCPTC and cPTC tissues were analyzed with the Limma package of R. The linear model fitting (lmFit) function in the Limma package was used to calculate fold changes, and empirical Bayes statistics (eBayes) were used to estimate standard errors. Only genes with |log2FC| > 1.0 and *P* < .05 were considered to be significantly DEGs. The R script used in Limma package is provided in Supplementary File 1. Hierarchical analysis of the identified DEGs was achieved by Cluster 3.0 (calculating average linkage based on Pearson correlation coefficient) and then visualized via the Java TreeView 1.16r4 (http://www.treeview.net).

### 2.3 Functional enrichment analysis

The Database for Annotation, Visualization, and Integrated Discovery (DAVID, https://david.ncifcrf.gov/) online system was utilized for candidate DEGs functions and pathway enrichment analyses. Gene Ontology analysis (GO, classification including biological process (BP), cellular component (CC) and molecular function (MF)) and Kyoto Encyclopedia of Genes and Genomes (KEGG) pathway enrichment analyses were performed. The cut-off was set at a *P* value < .05.

### 2.4 Protein–protein interaction network analysis

The PPI network was constructed using the Search Tool for the Retrieval of Interacting Genes/Proteins (STRING, https://string-db.org, the minimum required interaction score was set at 0.4) database, and the results were visualized with Cytoscape software 3.5.1. Subnetwork models were selected using the plugin molecular complex detection (MCODE) application in Cytoscape. The important coexpression modules of DEGs were detected from the PPI network, and the criteria for defining a module were an MCODE score ≥5 and a number of nodes ≥4.^[16]^ Functional enrichment of the genes in the subnetworks was performed with STRING. Hub genes were explored using the CytoHubba plugin application with the topological analysis of Maximal Clique Centrality (MCC, top 10 nodes ranked by MCC).^[17]^ Cytohubba is a tool that provides 11 topological analysis and 6 centralities based on shortest paths to detect the hub genes (key genes).^[18]^

### 2.5 Statistical analysis

Statistical analysis was carried out using SPSS 21.0 and R studio. The Chi-squared or Fisher exact test was used to analyze the associations between different histological diagnoses and clinicopathological parameters. Kaplan–Meier and log-rank tests were applied to illustrate the risk of disease-free and overall survival rates between patients with different pathologic types. A *P* value < .05 was considered statistically significant. ROC curves and the area under the curve (AUC) were determined to test the specificity and sensitivity of each candidate gene for diagnostic predictions. ROC analysis was used to find a cut-off point of each hub gene for distinguishing different pathologic types.

## 3 Results

### 3.1 Somatic mutations in TCPTC and cPTC

**Figure 1:**
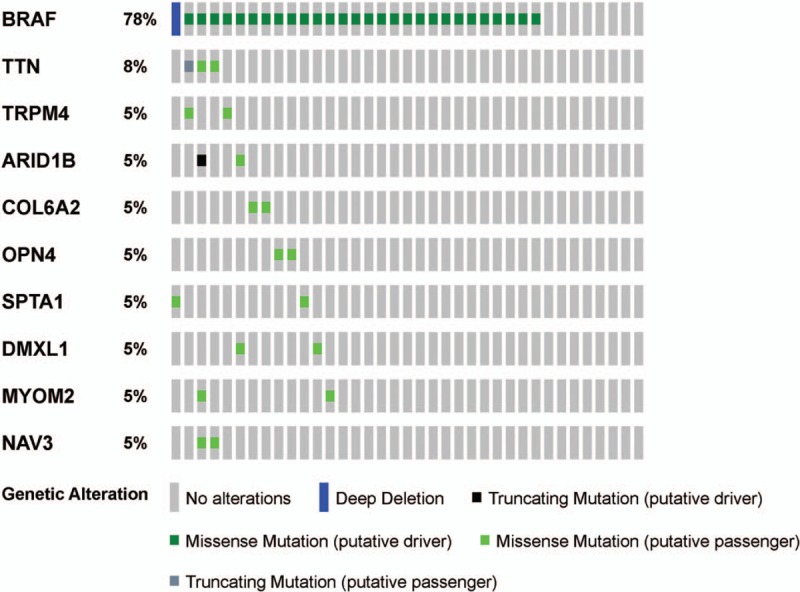
OncoPrint of mutated genes in TCPTC. Ten altered genes (mutation in more than 2 cases) in patients with TCPTC are presented. BRAF mutation is the predominant mutation type, whereas other gene mutation types occurred at a much lower rate. cPTC = classical/conventional variant Papillary Thyroid Carcinoma, TCPTC = tall cell variant papillary thyroid carcinoma.

The gene mutation frequencies in patients with TCPTC and cPTC were obtained by selecting relevant case IDs through the cBioPortal website. In total, altered genes (mutations in more than 2 cases) occurred in 29 of the 37 TCPTC cases. The top 10 gene mutation profiles are shown in Figure 1. Alterations in *BRAF* (28 *BRAF* V600E mutations and 1 deletion mutation) were particularly prevalent in TCPTC with a mutation frequency of 78%. Patients with the other 9 gene mutations also harbored *BRAF* mutations. None of the total 37 patients harbored RAS gene mutations. *BRAF* mutation was also the predominant mutation type in cPTC (occurring in 56% of cPTC patients, Supplementary Fig. S1), followed by RET fusion (with a relatively low occurrence rate of 7%) and RAS mutations (including NRAS, HRAS and KRAS mutations with frequencies of 4%, 1.7%, and 0.71%, respectively).

### 3.2 TCPTC was associated with aggressive clinicopathological parameters

**Figure 2:**
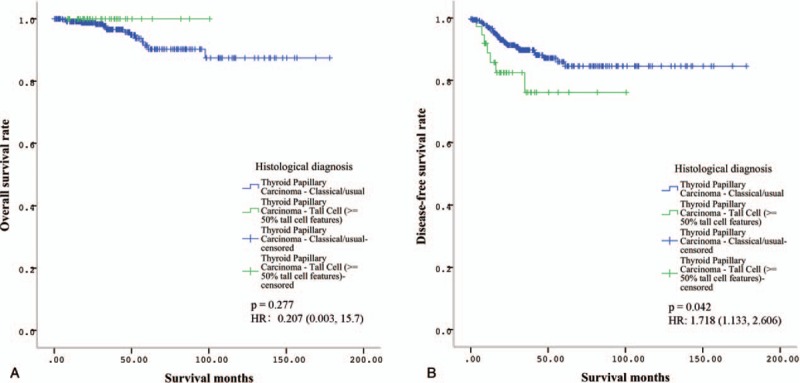
Kaplan–Meier survival analyses of patients with TCPTC and cPTC. A. No difference in the overall survival rate was evident between the patients with TCPTC and those with cPTC. B. Disease-free survival analysis revealed that patients with TCPTC had a poorer disease-free survival rate. HR = hazards ratios. cPTC = classical/conventional variant Papillary Thyroid Carcinoma, TCPTC = tall cell variant papillary thyroid carcinoma.

To address the clinical differences between TCPTC and cPTC, the correlations of the two histological diagnoses with clinicopathological parameters were examined. The results showed that TCPTC was significantly correlated with a patient age >45 years, tumor multifocality, extrathyroidal extension, a higher T stage, advanced AJCC TNM stages, and *BRAF* V600E mutation. No significance was observed for patient gender, N stage or M stage (Table 1). Additionally, Kaplan–Meier analysis revealed that patients with TCPTC had a poorer disease-free survival rate, whereas no difference in the overall survival rate was evident between the TCPTC and cPTC patients (Fig. 2). These results suggested that TCPTC patients had more aggressive clinicopathological features and poorer outcomes than cPTC patients.

**Table 1:**
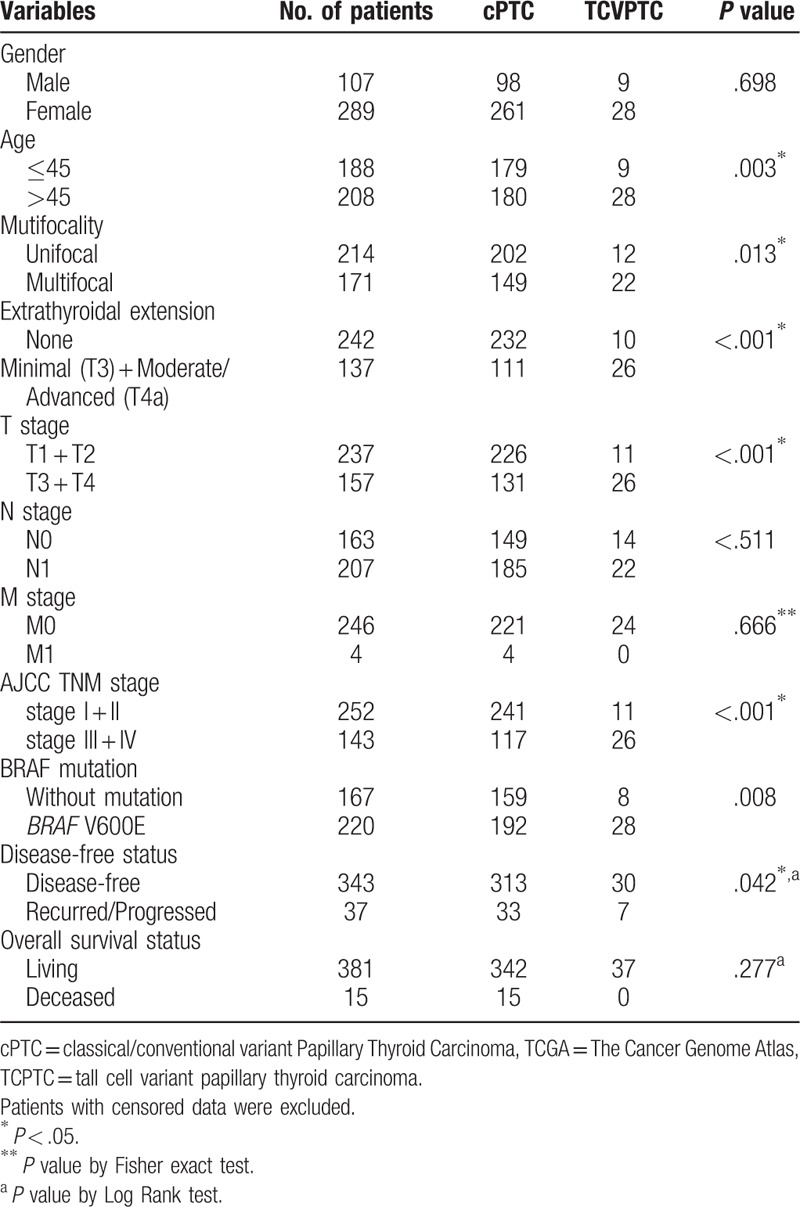
Clinicopathological features of TCPTC and cPTC patients in TCGA dataset.

### 3.3 TCPTC-specific differentially expressed mRNAs

**Figure 3:**
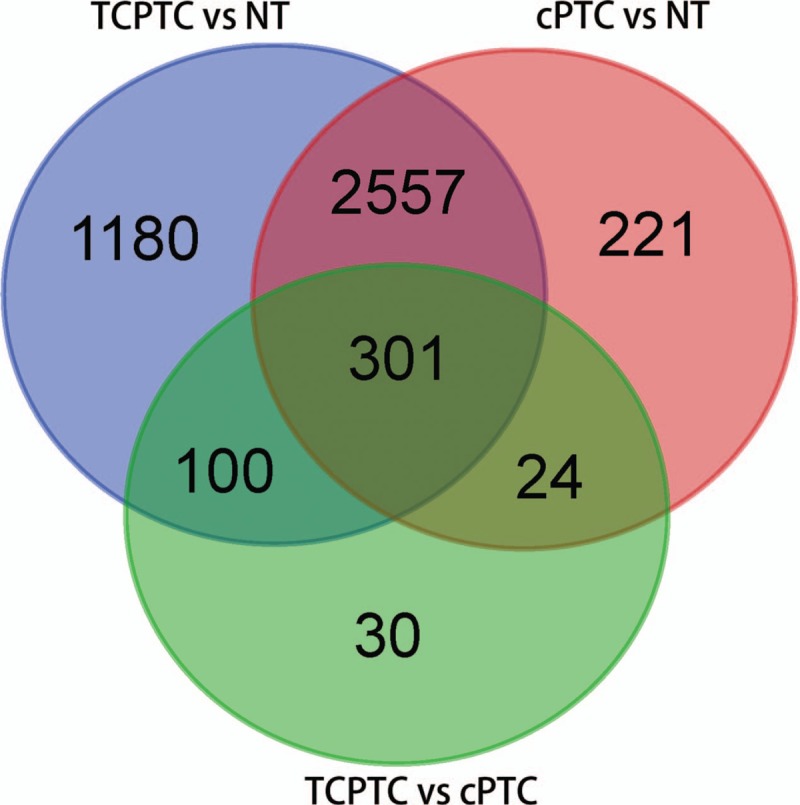
Venn diagram of 301 TCPTC-specific DEGs. A total of 301 TCPTC-specific DEGs were identified from the 3 comparison groups (TCPTC versus NT, cPTC versus NT, and TCPTC versus cPTC). DEGs = differentially expressed genes, NT = normal tissues, cPTC = classical/conventional variant Papillary Thyroid Carcinoma, TCPTC = tall cell variant papillary thyroid carcinoma.

**Figure 4:**
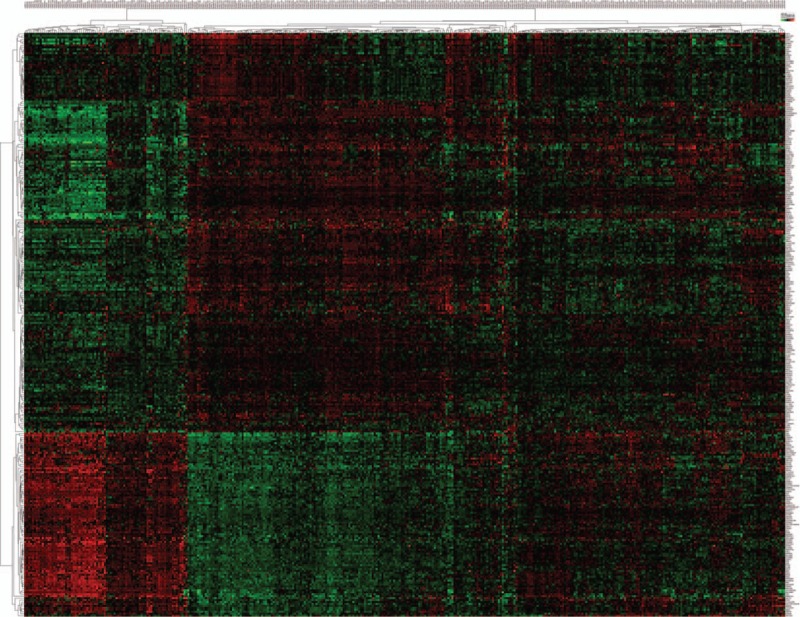
Heatmap of the 301 TCPTC-specific DEGs. Hierarchical analysis of the 301 TCPTC-specific DEGs based on their expression values in the TCPTC and cPTC tissues. All values are presented as log10 (RSEM), DEGs = differentially expressed genes, cPTC = classical/conventional variant Papillary Thyroid Carcinoma, RSEM = RNAseq by expectation-maximization, TCPTC = tall cell variant papillary thyroid carcinoma.

Using |log2FC| > 1.0 and *P* < .05 as the cut-off criteria, we extracted 4138 DEGs (2312 downregulated and 1826 upregulated genes) between the TCPTC and normal tissues, and 3103 DEGs (1723 downregulated and 1380 upregulated genes) between the cPTC and normal tissues. The DEGs between the TCPTC and normal tissues were defined as TCPTC-related DEGs. Among the TCPTC-related DEGs, *TMPRSS6*, *TM7SF4*, *SYT12*, *MUC21*, and *TMPRSS4* were the most significantly upregulated DEGs, and *PKHD1L1*, *TFF3*, *LRP1B*, *CUX2*, and *ZMAT4* were the most significantly downregulated DEGs. Furthermore, 455 DEGs were identified between TCPTC and cPTC with the same threshold, of which 134 genes were downregulated and 321 genes were upregulated. DEGs between TCPTC and cPTC that were also found among the PTC-related DEGs (genes dysregulated in both TCPTC and cPTC tissues compared with normal tissues) were defined as TCPTC-specific DEGs. According to this protocol, 301 TCPTC-specific DEGs, including 96 downregulated and 205 upregulated genes, were identified between the TCPTC and cPTC tissues (Figs. 3 and 4). The average expression value in the PTC tissues (including TCPTC and cPTC tissues), fold change and *P* value details of the dysregulated genes are shown in Supplementary Table S2. *COL11A1*, *MMP13*, *VTCN1*, *COL10A1*, and *SLC18A3* were the most significantly upregulated DEGs in the TCPTC tissues, while *CA4*, *EDN3*, *TPO*, *PKHD1L1* and *FOXJ1* were the most significantly downregulated DEGs in the TCPTC tissues compared with the cPTC tissues. Interestingly, the expression levels of nearly all putative oncogenes (genes upregulated in both TCPTC and cPTC compared with normal tissues, except for 7 genes) were much higher in the TCPTC tissues than those in the cPTC tissues. The expression levels of nearly all putative tumor suppressors (genes downregulated both in TCPTC and cPTC compared with normal tissues, except for 1 gene) were much lower in the TCPTC tissues than those in the cPTC tissues.

### 3.4 GO and KEGG pathway enrichment analysis of TCPTC-related and TCPTC-specific DEGs

**Figure 5:**
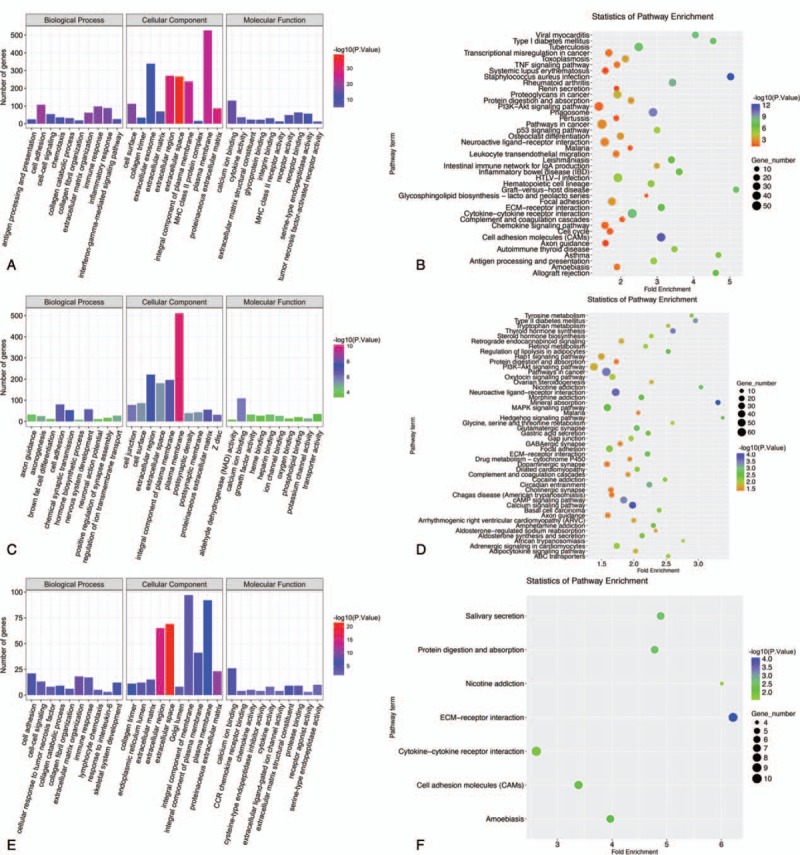
GO and KEGG pathway enrichment analyses of TCPTC DEGs. A. GO annotation for TCPTC-related upregulated DEGs. GO biological process, cellular component and molecular function terms, the number of enriched genes and –log10 (*P* value) is presented. B. KEGG pathway enrichment analysis of TCPTC-related upregulated DEGs. The pathway terms, enriched genes numbers, –log10 (*P* value) and fold enrichment scores are presented. C. GO annotation for TCPTC-related downregulated DEGs. D. KEGG Pathway enrichment analysis of TCPTC-related downregulated DEGs. E. GO annotation for TCPTC-specific DEGs. F. KEGG pathway enrichment analysis of TCPTC-specific DEGs. DEGs = differentially expressed genes, GO = gene ontology, KEGG = kyoto encyclopedia of genes and genomes, TCPTC = tall cell variant papillary thyroid carcinoma.

To gain a better understanding of the gene functions and signaling pathways of TCPTC-related and TCPTC-specific DEGs, online GO and KEGG pathway enrichment analyses were conducted using DAVID. Due to gene quantitative restriction, we enriched the upregulated genes and the downregulated TCPTC-related genes separately. For the TCPTC-related upregulated DEGs, the GO analysis results showed that the DEGs were significantly enriched in cell adhesion at the BP level, in integral component of membrane at the CC level and in calcium ion binding at the MF level. The top 10 enriched terms are shown in Figure 5A. Furthermore, the enriched KEGG pathways of the TCPTC-related upregulated DEGs included pathways in cancer, cytokine–cytokine receptor interactions, HTLV-I infection, PI3K-Akt signaling, and cell adhesion (Fig. 5B). For the TCPTC-related downregulated DEGs, the GO analysis results showed that the DEGs were significantly enriched in chemical synaptic transmission at the BP level, plasma membrane at the CC level, and calcium ion binding at the MF level (Fig. 5C). Furthermore, the enriched KEGG pathways of the TCPTC-related downregulated DEGs included pathways in cancer, neuroactive ligand-receptor interactions, PI3K-Akt signaling, MAPK signaling, and cAMP signaling (Fig. 5D). For the TCPTC-specific DEGs, the top enriched GO terms for BP, CC and MF were extracellular matrix organization, extracellular space, and extracellular matrix structural constituent, respectively (Fig. 5E). The enriched KEGG pathways of the TCPTC-specific DEGs included pathways in cytokine-cytokine receptor interactions, ECM-receptor interaction, neuroactive ligand-receptor interaction, cell adhesion molecules, and focal adhesions (Fig. 5F).

### 3.5 PPI network construction and hub gene selection

**Figure 6:**
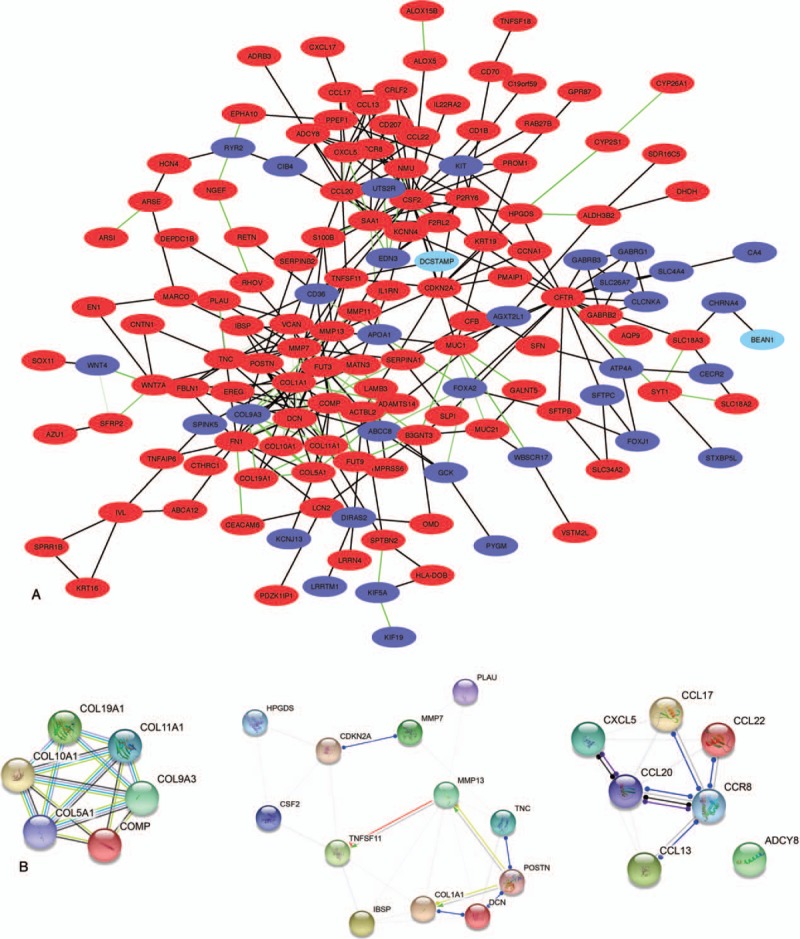
The PPI network of the TCPTC-specific DEGs. A. The network of 301 TCPTC-specific DEGs includes 166 nodules and 321 edges. The red nodules represent the upregulated genes, and the blue nodules represent the downregulated genes. Green edges indicate a combined score of interactive genes >0.9. B. The 3 subnetworks identified by the MCODE application in Cytoscape (networks were constructed through the STRING database). DEGs = Differentially expressed genes, MCODE = plugin molecular complex detection, PPI = protein-protein interaction, TCPTC = tall cell variant papillary thyroid carcinoma.

To identify TCPTC-specific biomarkers, a PPI network was constructed with only 301 TCPTC-specific DEGs by the STRING database and then visualized with Cytoscape software. The PPI network consisted of 166 nodes and 321 edges (Fig. 6A). A total of 3 subnetworks, comprising 25 nodes and 76 edges, were found using the defined criteria (Fig. 6B). GO enrichment revealed that these genes were annotated in extracellular matrix organization at the BP level, extracellular space at the CC level, and extracellular matrix structural constituent at the MF level. The enriched KEGG pathways included pathways in cytokine-cytokine receptor interactions, ECM-receptor interactions, chemokine signaling, focal adhesion, and PI3K-Akt signaling. All enriched GO and KEGG terms are shown in Supplementary Table S3. Furthermore, 10 candidate hub genes (including *COL9A3*, *COL5A1*, *COL1A1*, *COL10A1*, *COL11A1*, *SAA1*, *CCL20*, *NMU*, *CCR8*, and *CXCL5*) were identified from the total PPI network. All candidate hub genes except for *NMU* and *SAA1* were among the genes involved in the top subnetworks. Among these hub genes, 7 (including, *COL5A1*, *COL1A1*, *COL10A1*, *COL11A1*, *CCL20*, *CCR8*, and *CXCL5*) were upregulated in both TCPTC and cPTC compared with the normal tissues, and only 1 gene (*COL9A3*) was downregulated. Together, these results suggest that these 8 genes may play a significant role in the progression of TCPTC. The enriched GO and KEGG terms for these genes are shown in Supplementary Table S3.

### 3.6 Diagnostic value of hub genes in TCPTC

**Figure 7:**
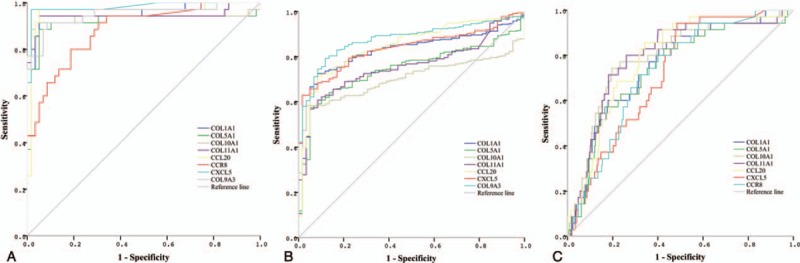
ROC curves for the diagnostic performances of the hub genes. A. Diagnostic performance of the hub genes for differentiating TCPTC from normal tissues. All 8 hub genes showed high diagnostic accuracy. B. Diagnostic performance of the hub genes for differentiating cPTC from normal tissues. Seven hub genes could be used to distinguish cPTC from normal tissues. C. Diagnostic performance of the hub genes for differentiating TCPTC from cPTC tissues. All 7 upregulated hub genes showed high diagnostic accuracy. cPTC = classical/conventional variant Papillary Thyroid Carcinoma, ROC = receiver operating characteristic, TCPTC = tall cell variant papillary thyroid carcinoma.

ROC analyses were performed to evaluate the specificities and sensitivities of the hub genes for diagnostic predictions. The diagnostic performances were carried out in 3 independent groups (namely, TCPTC vs. normal tissues, cPTC vs. normal tissues, and TCPTC vs. cPTC tissues). To determine the discriminatory abilities of these candidate genes in each group, an AUC > 0.7 was fixed as the threshold. The ROC curve analysis demonstrated that all 8 hub genes yielded high diagnostic accuracy in discriminating TCPTC from normal tissues (Fig. 7A). Similarly, except for CCR8, the aforementioned hub genes could distinguish cPTC from normal tissues (Fig. 7B). Furthermore, all 7 upregulated hub genes could robustly distinguish TCPTC from cPTC tissues (Fig. 7C). Collectively, 6 hub genes (*COL5A1*, *COL1A1*, *COL10A1*, *COL11A1*, *CCL20*, and *CXCL5*) were identified in all 3 independent groups. The AUCs and cut-off values of these 6 genes for distinguishing the 3 independent groups are presented in Table 2. Our results suggest that a combination of these hub genes can be used not only for the differential diagnosis of PTC, including TCPTC and cPTC, from normal samples, but also for the differential diagnosis of TCPTC from cPTC samples.

**Table 2:**
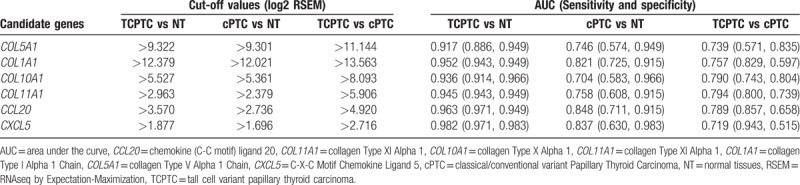
Cut-off values and AUCs of 6 genes for distinguishing the 3 independent groups.

## 4 Discussion

In this study, we studied the gene mutation patterns and clinical characteristics of TCPTC by utilizing data obtained from TCGA–THCA dataset. Multi-group analyses were performed to identify aberrantly expressed TCPTC-related and TCPTC-specific genes, and these analyses were followed by functional and pathway enrichment analyses. Importantly, we highlighted the diagnostic values of several hub genes not only for distinguishing TCPTC and cPTC from normal tissues, but also for distinguishing TCPTC from cPTC tissues. By analyzing the TCGA-THCA data, Stokowy et al revealed molecular differences, including microRNA and mRNA differences, between TCPTC and FVPTC.^[19]^ To the best of our knowledge, this investigation is the first global study of gene mutations, screening for TCPTC-specific DEGs, and diagnostic approaches for candidate biomarkers of TCPTC by utilizing TCGA-THCA data.

We found that *BRAF* V600E was the predominant gene mutation type with a high incidence rate (78%), whereas other mutation types were rare in patients with TCPTC. *BRAF* V600E was also the predominant gene mutation type, and other gene mutation types, including RET fusion and RAS mutations, were also common in patients with cPTC. Consistent with other reports, *BRAF* V600E-positive PTC was often the conventional or tall cell variant.^[20]^*BRAF* mutation was more frequent in TCPTC than that in cPTC, and more frequent in tall cell variant papillary thyroid microcarcinoma (PTMC) than that in classic PTMC.^[21,22]^ Some rare gene mutations, such as the Titin (*TTN*) mutation, have been observed in TCPTC. A truncating *TTN* mutation has been identified as a cause of congenital myopathy that is reported as centronuclear myopathy,^[23]^ but no evidence has shown that the *TTN* mutation is associated with cancers. Some rare gene mutation types that were not identified in our study, including the RET/PTC rearrangement and TERT promoter mutation were also reported to be prevalent in TCPTC.^[24,25]^ We disclosed that TCPTC was associated with more aggressive clinicopathological parameters and poorer outcomes than cPTC. This finding is consistent with the results of the other studies. The overall survival analysis showed a better survival trend of patients with TCPTC compared to those with cPTC. This may be related to the number of TCPTC cases and the duration of follow-up time. A large multicenter study launched by Shi et al demonstrated the differential prognostic risk of the 3 major PTC variants and established a unique risk order of TCPTC > cPTC >> FVPTC.^[1]^ A recent meta-analysis also revealed that TCPTC was associated with more aggressive clinicopathological characteristics and poorer prognoses.^[10]^ The percentage of TC necessary to diagnose TCPTC has been debated. Beninato et al revealed that the aggressive features conferred by the presence of TC in PTCs occurred with as little as 10% TC and were maintained with increasing percentages.^[26]^ Oh et al revealed that PTC with 10% to 50% TC showed a similar *BRAF* mutation rate and clinicopathological features to TCPTC.^[27]^ Ganly et al suggested that consideration should be given to using a 30% TC threshold to diagnose TCPTC,^[11]^ whereas Ito et al suggested that diagnosing TCPTC with a TC threshold ≥50% is appropriate.^[28]^ The TC percentage was ≥50% in all of the TCPTC patients included in this study. These results suggested that the high incidence rate of *BRAF* mutation and TC percentage partly explained the aggressive features of TCPTC.

Matriptase-2 (TMPRSS6) is a member of the type 2 transmembrane serine protease family. TMPRSS6 has been implicated in the progression of cancers, including breast, prostate, and colorectal cancers.^[29]^ Strong TMRRSS6 expression has been detected in thyroid tissues,^[30]^ which may indicate that TMRRSS6 plays a specialized role in PTC. COL11A1 dysregulation across cancer types is particularly striking, including colorectal, ovarian, breast, head and neck, lung, and brain cancers.^[31]^ This result indicates that COL11A1 may serve as a remarkable biomarker for various types of cancers and as a target for cancer therapy. Park et al revealed that the COL11A1 gene might be associated with PTC and, in particular, that the T allele of rs1763347 and rs2229783 might contribute to reducing the risk of PTC.^[32]^ Carbonic anhydrase 4 (CA4) is a member of the carbonic anhydrases family. Davidov conducted a tissue microarray study that comprised 26 follicular thyroid carcinomas and 53 follicular adenomas from patients with indeterminate thyroid nodules. By analyzing the staining results for 17 immunohistochemical biomarkers, the authors revealed that loss of immunoreactivity of CA4 was associated with malignancy in indeterminate thyroid specimens.^[33]^ These genes are the topmost dysregulated genes among the TCPTC-related and TCPTC-specific DEGs. However, the functions and specific mechanisms of these genes need elucidation. GO and KEGG pathway analyses of the DEGs were performed to understand the gene functions and interactions of the TCPTC-related and TCPTC-specific DEGs. The enriched KEGG pathways of the TCPTC-related DEGs included typical pathways involved in thyroid cancer, such as the MAPK and PI3K-Akt signaling pathways.^[34–37]^ These data suggest that TCPTC shares some same signaling pathway abnormalities with cPTC. Intriguingly, the results of our study showed that the dysregulated levels of both putative oncogenes and tumor suppressors in TCPTC were higher than those in cPTC. Furthermore, the higher degree of abnormality of the enriched functions and signaling pathways associated with the TCPTC-specific DEGs may be one reason for the higher degree of malignancy of TCPTC.

TCPTC is usually associated with a poor outcome, and the correct pre-surgery recognition of this variant is important for clinical management. Evranos et al conducted a retrospective study to compare the malignancy rates determined by fine needle aspiration (FNA) and histopathological results. Pre-surgical cytology showed malignancy or suspicion of malignancy in 92% of the aggressive (including TCPTC) variants of histopathologically confirmed PTC.^[38]^ These results suggest that the Bethesda classification is a reliable indicator of the malignancy of nodules with aggressive variant PTC. However, other studies have revealed that pathologists often face the dilemma of a proper diagnosis of TCPTC, based on cytology as well as histology.^[13]^ A few studies have described the identification of biomarkers to improve the differential diagnosis of TCPTC from cPTC lesions.^[39,40]^ For example, Nardone et al have revealed that TCPTC specimens expressed significantly greater levels of c-Met than other forms of PTC and benign thyroid disease.^[40]^ In this study, we revealed for the first time that several biomarkers showed excellent predictive values not only for the differential diagnosis of TCPTC from normal tissues but also for the differential diagnosis of TCPTC from cPTC tissues. Future studies with more cost-effective approaches through FNA or postoperative histopathologies, such as RT-PCR and immunohistochemical staining, should be conducted instead of RNA sequencing to validate the clinical utility of the individual or combined candidate biomarkers identified in our study.

In summary, we have identified notable alterations of TCPTC, including gene mutations, DEGs and related pathways, and evaluated the diagnostic performances of hub genes for TCPTC. We disclosed that TCPTC was associated with more aggressive clinicopathological parameters and poorer outcomes than cPTC. This could be partly attributed to the high incidence rate of *BRAF* mutation. Our work may provide additional diagnostic targets for TCPTC. The use of molecular profiles for differential diagnoses of TCPTC from cPTC should be evaluated by studies with larger patient cohorts. More work is needed to elucidate the functional mechanisms of these genes and to validate the clinical utility of these biomarkers with more cost-effective approaches.

## Author contributions

Xia F and Li X. conception and design; All authors. methodology and development; Xia F, Jiang B, Chen Y, and Li X. data acquisition and analysis; Xia F and Li X. manuscript preparation and editing.

**Conceptualization:** Fada Xia and Xinying Li.

**Data curation:** Fada Xia, Bo Jiang, Yong Chen, Xin Du, and Xinying Li.

**Formal analysis:** Fada Xia, Bo Jiang, Xin Du, Yao Peng, and Xinying Li.

**Funding acquisition:** Xinying Li.

**Investigation:** Xinying Li.

**Methodology:** Fada Xia, Bo Jiang, Yong Chen, Wenlong Wang, Zhuolu Wang, and Xinying Li.

**Project administration:** Xinying Li.

**Supervision:** Xinying Li.

**Writing – original draft:** Fada Xia.

**Writing – review & editing:** Xinying Li.

## Supplementary Material

Supplemental Digital Content
