# Supplementary material for: Prediction of novel target genes and pathways involved in tall cell variant papillary thyroid carcinoma
Source: Medicine (Baltimore). 2018 Dec 21;97(51):e13802. doi: 10.1097/MD.0000000000013802 (PMC6319788; doi:10.1097/MD.0000000000013802)
Supplement: Supplemental Digital Content [file medi-97-e13802-s001.pdf]

Supplementary File 1. The R script used in Limma package for identification of DEGs.

The R script used in Limma package for identification of DEGs.

```
path = "/Users/*/*"
setwd(path)

options(max.print=1000000)

dat = read.table("expressionvalues.txt",header=T,sep="\t")
View(head(dat))
dim(dat)

dat1 = dat[,-1]
rownames(dat1) = dat[,1]
View(head(dat1))

write.table(dat.1,"datExp.txt",col.names = T,row.names = T,quote=F,sep="\t")

dat.exp = read.table("datExp.txt",header=T,sep="\t")
View(head(dat.exp))

dat.exp[dat.exp == 0] = 1
dat.exp = log(dat.exp,2)
View(dat.exp)

pheno = NULL
pheno$sample = colnames(dat.exp)
pheno$group = c(rep('tumor',ncol(dat.tumor)),rep('normal',ncol(dat.normal)))
pheno = as.data.frame(pheno)
head(pheno)
```

```
library(limma)
Group = factor(pheno$group,levels=c('tumor','normal'))
design = model.matrix(~0+Group)
colnames(design) <- c('tumor','normal')
design

fit <- lmFit(dat.exp, design)
contrast.matrix <- makeContrasts(tumor-normal,#1
                                 levels=design)
fit2 <- contrasts.fit(fit, contrast.matrix)
fit2 <- eBayes(fit2)
all.deg = NULL
diff = topTable(fit2,adjust.method="fdr",coef=1,p.value=0.05,
                lfc=log(2,2),number=50000,sort.by = 'logFC')
write.table(diff,'DEG.txt',col.names=T,row.names=T,quote=F,sep="\t")
```

#pheno$group can be changed when identifying different compared groups (TCPTC vs cPTC, TCPTC or cPTC vs Normal).

Supplementary Figure S1. OncoPrint of the top 20 mutated genes in the patients with cPTC. BRAF mutation is also the predominant mutation type in cPTC, followed by RET fusion and RAS mutations.

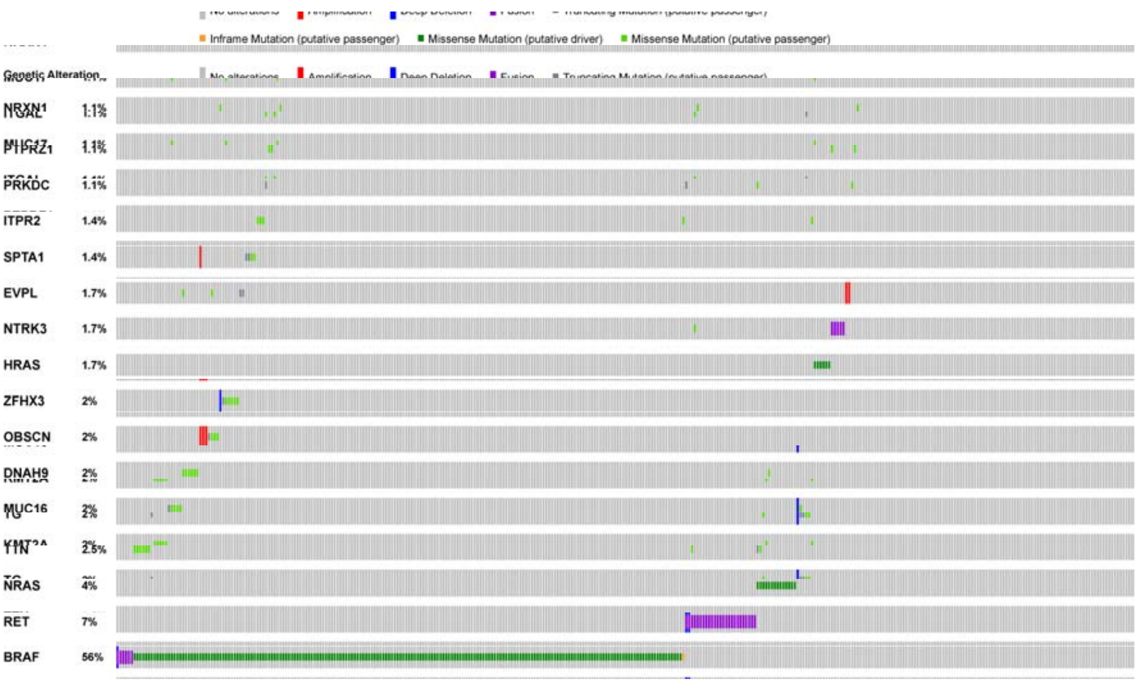

Inframe Mutation (putative passenger)
Missense Mutation (putative driver)
Missense Mutation (putative passenger)
Genetic Alteration
No alterations
Amplification
Deep Deletion
Fusion
Truncating Mutation (putative passenger)
NRXN1 1.1%
MUC17 1.1%
PRKDC 1.1%
ITPR2 1.4%
SPTA1 1.4%
EVPL 1.7%
NTRK3 1.7%
HRAS 1.7%
ZFHX3 2%
OBSCN 2%
DNAH9 2%
MUC16 2%
TTN 2.5%
NRAS 4%
RET 7%
BRAF 56%

Supplementary Table 1: TCGA sample IDs of 357 cPTC and 35 tall cell variant PTC patients with mRNA sequence and clinical data.

357 cPTC sample IDs

Sample IDs	Sample IDs	Sample IDs	Sample IDs
TCGA-4C-A93U	TCGA-DJ-A3US	TCGA-EL-A3T7	TCGA-ET-A3BU
TCGA-BJ-A0YZ	TCGA-DJ-A3UU	TCGA-EL-A3T8	TCGA-ET-A3BV
TCGA-BJ-A0Z0	TCGA-DJ-A3UW	TCGA-EL-A3T9	TCGA-ET-A3BW
TCGA-BJ-A0Z3	TCGA-DJ-A3UX	TCGA-EL-A3TA	TCGA-ET-A3BX
TCGA-BJ-A0Z5	TCGA-DJ-A3UY	TCGA-EL-A3TB	TCGA-ET-A3DO
TCGA-BJ-A0ZA	TCGA-DJ-A3UZ	TCGA-EL-A3ZG	TCGA-ET-A3DP
TCGA-BJ-A0ZB	TCGA-DJ-A3V2	TCGA-EL-A3ZH	TCGA-ET-A3DR
TCGA-BJ-A0ZC	TCGA-DJ-A3V3	TCGA-EL-A3ZK	TCGA-ET-A3DS
TCGA-BJ-A0ZE	TCGA-DJ-A3V9	TCGA-EL-A3ZL	TCGA-ET-A3DT
TCGA-BJ-A0ZJ	TCGA-DJ-A3VA	TCGA-EL-A3ZM	TCGA-ET-A3DU
TCGA-BJ-A18Y	TCGA-DJ-A3VD	TCGA-EL-A3ZN	TCGA-ET-A40S
TCGA-BJ-A18Z	TCGA-DJ-A3VE	TCGA-EL-A3ZO	TCGA-ET-A40T
TCGA-BJ-A190	TCGA-DJ-A3VI	TCGA-EL-A3ZP	TCGA-FE-A230
TCGA-BJ-A192	TCGA-DJ-A4UL	TCGA-EL-A3ZQ	TCGA-FE-A231
TCGA-BJ-A28R	TCGA-DJ-A4UP	TCGA-EL-A3ZR	TCGA-FE-A232
TCGA-BJ-A28S	TCGA-DJ-A4UT	TCGA-EL-A3ZS	TCGA-FE-A233
TCGA-BJ-A28T	TCGA-DJ-A4V0	TCGA-EL-A3ZT	TCGA-FE-A234
TCGA-BJ-A28V	TCGA-DJ-A4V2	TCGA-EL-A4JV	TCGA-FE-A235
TCGA-BJ-A28W	TCGA-DJ-A4V5	TCGA-EL-A4JW	TCGA-FE-A236
TCGA-BJ-A28Z	TCGA-DO-A1K0	TCGA-EL-A4JX	TCGA-FE-A237
TCGA-BJ-A291	TCGA-DO-A2HM	TCGA-EL-A4JZ	TCGA-FE-A238
TCGA-BJ-A2N7	TCGA-E3-A3E0	TCGA-EL-A4K0	TCGA-FE-A239
TCGA-BJ-A2N8	TCGA-E3-A3E1	TCGA-EL-A4K1	TCGA-FE-A23A
TCGA-BJ-A2N9	TCGA-E3-A3E3	TCGA-EL-A4K2	TCGA-FE-A3PB
TCGA-BJ-A2NA	TCGA-E3-A3E5	TCGA-EL-A4K4	TCGA-FE-A3PC
TCGA-BJ-A2P4	TCGA-E8-A242	TCGA-EL-A4K6	TCGA-FK-A3SB
TCGA-BJ-A3EZ	TCGA-E8-A2EA	TCGA-EL-A4K7	TCGA-FK-A3SD
TCGA-BJ-A3F0	TCGA-E8-A2JQ	TCGA-EL-A4K9	TCGA-FK-A3SE

TCGA-BJ-A3PR	TCGA-E8-A3X7	TCGA-EL-A4KD	TCGA-FK-A3SG
TCGA-BJ-A3PT	TCGA-E8-A413	TCGA-EL-A4KG	TCGA-FK-A3SH
TCGA-BJ-A3PU	TCGA-E8-A414	TCGA-EL-A4KH	TCGA-FK-A4UB
TCGA-BJ-A45C	TCGA-E8-A415	TCGA-EL-A4KI	TCGA-FY-A2QD
TCGA-BJ-A45E	TCGA-E8-A416	TCGA-EM-A1CS	TCGA-FY-A3BL
TCGA-BJ-A45F	TCGA-E8-A417	TCGA-EM-A1CT	TCGA-FY-A3I4
TCGA-BJ-A45H	TCGA-E8-A418	TCGA-EM-A1CU	TCGA-FY-A3NM
TCGA-BJ-A45K	TCGA-E8-A419	TCGA-EM-A1CV	TCGA-FY-A3NN
TCGA-BJ-A4O8	TCGA-E8-A432	TCGA-EM-A22I	TCGA-FY-A3ON
TCGA-CE-A13K	TCGA-E8-A433	TCGA-EM-A22K	TCGA-FY-A3R6
TCGA-CE-A27D	TCGA-E8-A434	TCGA-EM-A22M	TCGA-FY-A3R7
TCGA-CE-A3MD	TCGA-E8-A436	TCGA-EM-A22O	TCGA-FY-A3R8
TCGA-CE-A3ME	TCGA-E8-A437	TCGA-EM-A22P	TCGA-FY-A3R9
TCGA-CE-A481	TCGA-E8-A438	TCGA-EM-A2CS	TCGA-FY-A3RA
TCGA-CE-A482	TCGA-E8-A44K	TCGA-EM-A2OX	TCGA-FY-A3TY
TCGA-CE-A483	TCGA-E8-A44M	TCGA-EM-A2OZ	TCGA-FY-A3YR
TCGA-CE-A484	TCGA-EL-A3CL	TCGA-EM-A2P0	TCGA-FY-A40K
TCGA-CE-A485	TCGA-EL-A3CM	TCGA-EM-A2P1	TCGA-FY-A40L
TCGA-DE-A0XZ	TCGA-EL-A3CN	TCGA-EM-A2P3	TCGA-FY-A40M
TCGA-DE-A0Y2	TCGA-EL-A3CO	TCGA-EM-A3AK	TCGA-FY-A4B0
TCGA-DE-A0Y3	TCGA-EL-A3CR	TCGA-EM-A3AN	TCGA-FY-A4B3
TCGA-DE-A3KN	TCGA-EL-A3CS	TCGA-EM-A3AO	TCGA-FY-A4B4
TCGA-DE-A4M8	TCGA-EL-A3CT	TCGA-EM-A3AQ	TCGA-FY-A76V
TCGA-DE-A4M9	TCGA-EL-A3CU	TCGA-EM-A3AR	TCGA-GE-A2C6
TCGA-DE-A4MA	TCGA-EL-A3CV	TCGA-EM-A3FJ	TCGA-H2-A26U
TCGA-DE-A4MB	TCGA-EL-A3CW	TCGA-EM-A3FK	TCGA-H2-A2K9
TCGA-DE-A4MC	TCGA-EL-A3CX	TCGA-EM-A3FM	TCGA-H2-A3RI
TCGA-DE-A4MD	TCGA-EL-A3CY	TCGA-EM-A3FO	TCGA-H2-A421
TCGA-DE-A69J	TCGA-EL-A3CZ	TCGA-EM-A3FQ	TCGA-H2-A422
TCGA-DE-A69K	TCGA-EL-A3D0	TCGA-EM-A3FR	TCGA-IM-A3EB
TCGA-DE-A7U5	TCGA-EL-A3D1	TCGA-EM-A3O3	TCGA-IM-A3ED
TCGA-DJ-A13O	TCGA-EL-A3D4	TCGA-EM-A3O7	TCGA-IM-A420
TCGA-DJ-A13P	TCGA-EL-A3D5	TCGA-EM-A3SU	TCGA-J8-A3NZ

TCGA-DJ-A13T	TCGA-EL-A3D6	TCGA-EM-A3SX	TCGA-J8-A3O0
TCGA-DJ-A13U	TCGA-EL-A3GO	TCGA-EM-A3SZ	TCGA-J8-A3O1
TCGA-DJ-A13V	TCGA-EL-A3GP	TCGA-EM-A4FF	TCGA-J8-A3O2
TCGA-DJ-A1QD	TCGA-EL-A3GR	TCGA-EM-A4FM	TCGA-J8-A3YD
TCGA-DJ-A1QE	TCGA-EL-A3GS	TCGA-EM-A4FN	TCGA-J8-A3YE
TCGA-DJ-A1QF	TCGA-EL-A3GU	TCGA-EM-A4FO	TCGA-J8-A3YF
TCGA-DJ-A1QI	TCGA-EL-A3GV	TCGA-EM-A4FV	TCGA-J8-A3YG
TCGA-DJ-A1QN	TCGA-EL-A3GW	TCGA-ET-A25J	TCGA-J8-A3YH
TCGA-DJ-A1QQ	TCGA-EL-A3GX	TCGA-ET-A25K	TCGA-J8-A42S
TCGA-DJ-A2PN	TCGA-EL-A3GY	TCGA-ET-A25M	TCGA-J8-A4HW
TCGA-DJ-A2PO	TCGA-EL-A3GZ	TCGA-ET-A25N	TCGA-J8-A4HY
TCGA-DJ-A2PQ	TCGA-EL-A3H1	TCGA-ET-A25O	TCGA-KS-A41F
TCGA-DJ-A2PR	TCGA-EL-A3H2	TCGA-ET-A25P	TCGA-KS-A41J
TCGA-DJ-A2PS	TCGA-EL-A3H3	TCGA-ET-A2MX	TCGA-KS-A4I1
TCGA-DJ-A2PU	TCGA-EL-A3H4	TCGA-ET-A2MY	TCGA-KS-A4I3
TCGA-DJ-A2PV	TCGA-EL-A3H5	TCGA-ET-A2MZ	TCGA-KS-A4I5
TCGA-DJ-A2PW	TCGA-EL-A3H7	TCGA-ET-A2N0	TCGA-KS-A4I7
TCGA-DJ-A2PZ	TCGA-EL-A3H8	TCGA-ET-A39J	TCGA-KS-A4I9
TCGA-DJ-A2Q0	TCGA-EL-A3MW	TCGA-ET-A39K	TCGA-KS-A4IB
TCGA-DJ-A2Q1	TCGA-EL-A3MX	TCGA-ET-A39L	TCGA-KS-A4IC
TCGA-DJ-A2Q4	TCGA-EL-A3MY	TCGA-ET-A39M	TCGA-L6-A4EP
TCGA-DJ-A2Q5	TCGA-EL-A3MZ	TCGA-ET-A39N	TCGA-L6-A4EQ
TCGA-DJ-A2Q6	TCGA-EL-A3N2	TCGA-ET-A39P	TCGA-MK-A4N6
TCGA-DJ-A2Q7	TCGA-EL-A3N3	TCGA-ET-A39R	TCGA-MK-A4N7
TCGA-DJ-A2QC	TCGA-EL-A3T0	TCGA-ET-A39S	TCGA-MK-A4N9
TCGA-DJ-A3UK	TCGA-EL-A3T1	TCGA-ET-A3BN	TCGA-QD-A8IV
TCGA-DJ-A3UM	TCGA-EL-A3T2	TCGA-ET-A3BP	
TCGA-DJ-A3UN	TCGA-EL-A3T3	TCGA-ET-A3BQ	
TCGA-DJ-A3UO	TCGA-EL-A3T6	TCGA-ET-A3BS	

35 tall cell variant PTC sample IDs

Sample IDs	Sample IDs	Sample IDs	Sample IDs
TCGA-BJ-A0Z9	TCGA-DJ-A2PT	TCGA-DJ-A3V8	TCGA-ET-A3BO
TCGA-BJ-A0ZH	TCGA-DJ-A2PY	TCGA-DJ-A3VB	TCGA-ET-A3BT
TCGA-BJ-A28X	TCGA-DJ-A2Q3	TCGA-DJ-A3VF	TCGA-ET-A3DW
TCGA-BJ-A290	TCGA-DJ-A2Q9	TCGA-DJ-A4UW	TCGA-ET-A40Q
TCGA-BJ-A45I	TCGA-DJ-A3UQ	TCGA-DJ-A4V4	TCGA-ET-A4KN
TCGA-BJ-A45J	TCGA-DJ-A3V0	TCGA-ET-A25G	TCGA-IM-A3U2
TCGA-DJ-A13L	TCGA-DJ-A3V4	TCGA-ET-A25L	TCGA-IM-A3U3
TCGA-DJ-A1QH	TCGA-DJ-A3V5	TCGA-ET-A25R	TCGA-L6-A4EU
TCGA-DJ-A1QO	TCGA-DJ-A3V7	TCGA-ET-A39O	

Supplementary Table 2. Average Expression values; fold change and p values of 301 TCPTC-specific DEGs from the three comparison groups (TCPTC versus cPTC tissues, TCPTC versus normal tissues, and cPTC versus normal tissues)

	Average Expression	TCPTC vs cPTC		TCPTC vs normal		cPTC vs normal	
Genename	(log2 RSEM)	logFC	adj.P.Val	logFC	adj.P.Val	logFC	adj.P.Val
COL11A1	4.320328743	4.018401445	3.26E-06	6.973672851	1.02E-24	2.955271406	2.63E-09
MMP13	2.304903159	3.597314254	1.71E-06	5.711421788	1.20E-23	2.114107535	5.72E-07
VTCN1	4.709527828	2.9403214	0.000141773	7.074526406	2.96E-31	4.134205006	9.05E-19
COL10A1	5.874990034	2.80217714	4.67E-05	4.245557583	2.81E-20	1.443380443	0.000282059
SLC18A3	2.367109323	2.736262009	7.15E-05	4.941958603	9.38E-22	2.205696594	2.34E-08
MMP7	6.573233658	2.710259896	0.000152514	5.778336597	1.56E-18	3.068076701	2.41E-12
SFRP2	8.553188932	2.669864426	0.000964924	1.035554736	0.030270249	-1.63430969	0.000896524
CD1A	5.299930361	2.636947489	8.96E-05	6.214090571	6.18E-26	3.577143081	1.40E-17
SFTPB	12.93550709	2.560261262	0.000975954	6.746901589	2.20E-27	4.186640327	1.20E-17
C2CD4A	6.018512625	2.549583266	7.15E-05	6.252363216	5.84E-32	3.70277995	1.04E-20
CST1	2.21893159	2.524702333	0.000440831	4.674808596	9.76E-18	2.150106264	2.88E-07
CD177	3.949632805	2.430512593	0.001948031	5.80611098	7.32E-21	3.375598387	1.47E-12
FRMD5	5.713423458	2.366387913	4.67E-05	5.578202702	1.88E-35	3.211814789	6.15E-20
CST2	3.923062646	2.353509596	0.0009027	6.388595329	2.86E-30	4.035085734	1.50E-20

CCL17	4.713596587	2.350740293	0.00027576	4.938641907	1.07E-18	2.587901614	7.80E-11
CHI3L1	11.57962833	2.303230235	0.001304987	6.540848184	4.69E-26	4.237617949	9.19E-21
CD207	5.593464797	2.268371993	0.000235671	5.365796717	1.43E-19	3.097424725	2.51E-15
TM7SF4	9.621974524	2.24768038	0.008618867	9.02763954	7.85E-37	6.77995916	1.85E-33
S100B	6.138893796	2.247677117	0.000221567	3.59768634	1.44E-20	1.350009222	0.000245816
CCL20	4.113516161	2.246214666	7.07E-05	5.331552853	1.18E-23	3.085338187	8.86E-19
ADCY8	5.599732716	2.237657353	0.002972288	7.794321428	9.11E-39	5.556664075	7.78E-31
SLC6A14	4.486387713	2.234871435	0.00066512	4.865470083	5.50E-21	2.630598648	4.56E-11
IVL	5.334242928	2.219252707	0.001580937	7.165641031	9.90E-36	4.946388323	1.77E-28
729884	4.129650128	2.180433035	0.001729691	6.316655744	8.19E-34	4.136222708	4.41E-22
PDZK1IP1	9.010334388	2.171632739	0.000625107	6.125850822	1.27E-28	3.954218083	1.94E-22
DMBX1	4.025497513	2.160147558	0.002171934	6.389629959	1.09E-38	4.229482401	3.16E-22
C1QL2	3.005059934	2.14443079	0.001830978	3.597070019	3.09E-10	1.452639229	0.00071721
MUC21	7.153179807	2.144136281	0.003565675	8.575211844	6.28E-36	6.431075563	2.35E-39
CLDN10	7.263770034	2.134897808	0.008460674	8.152739042	4.73E-40	6.017841234	9.51E-31
PPAPDC1A	2.689976643	2.133023725	7.15E-05	3.23264194	5.39E-18	1.099618214	0.000519985
CXCL17	7.208500541	2.125895552	0.000336326	5.897579309	3.57E-30	3.771683757	3.93E-23
CYP26A1	1.541700034	2.125775434	1.42E-05	3.609723199	1.43E-16	1.483947766	5.72E-08
LCN2	8.620002003	2.112942282	0.000817288	6.331849273	2.67E-28	4.218906991	5.56E-26

TMEM92	5.591057678	2.112349934	0.000819858	7.656012115	9.63E-45	5.543662182	2.46E-40
RAB27B	2.630724321	2.08967273	0.000440831	4.045918697	6.78E-21	1.956245967	4.34E-08
C19orf59	3.907424447	2.037102186	0.000544443	5.603176121	6.82E-30	3.566073936	3.94E-22
ARSI	3.7292533	2.017790849	0.000198282	3.765905531	2.01E-21	1.748114682	6.76E-08
TMPRSS4	9.334330474	2.003217678	0.003479956	8.508088747	3.74E-40	6.504871068	5.52E-44
PSG8	3.513680951	2.002044603	0.010677543	5.354595957	5.76E-21	3.352551353	7.75E-13
SLC28A3	2.448514808	1.982444735	0.000111899	3.50215178	4.03E-17	1.519707045	7.90E-07
TMPRSS6	8.83678605	1.981840957	0.013938075	9.110170178	9.56E-42	7.128329221	7.51E-41
RETN	1.772697873	1.968174445	0.000294649	3.717526271	1.44E-16	1.749351826	1.53E-08
SYT12	6.112867106	1.947051524	0.001367693	4.344833484	2.36E-29	2.397781959	6.63E-11
ST6GALNA	6.880431528	1.942484133	0.005799205	6.486008385	4.71E-36	4.543524252	1.49E-24
CEACAM6	6.543988402	1.938316051	0.007851541	6.609312564	1.43E-25	4.670996514	5.74E-24
ALOX5	9.895614602	1.937619337	0.00027576	4.849751753	5.22E-23	2.912132416	7.23E-18
CD1B	2.381524986	1.936322652	0.000241132	3.175283282	1.05E-11	1.23896063	0.000148399
WNT7A	1.843923405	1.90707828	0.000216785	3.487469718	8.12E-17	1.580391438	1.83E-07
B3GNT3	8.72660745	1.887902894	0.008209085	7.649820669	2.25E-35	5.761917776	1.66E-34
DSC3	5.667949112	1.883503537	0.006781485	5.451385832	1.12E-19	3.567882295	1.82E-16
POSTN	9.937592266	1.865499163	8.96E-05	3.739340789	1.50E-20	1.873841627	3.05E-11
MYBPH	3.890029313	1.857826494	0.00185329	5.492188443	2.02E-27	3.634361949	1.17E-22

APCDD1L	1.90668846	1.856389838	7.15E-05	3.561223233	9.40E-21	1.704833395	8.60E-10
SLC34A2	14.91639838	1.847503609	0.003815772	6.763098328	6.41E-45	4.91559472	2.56E-32
PROM1	4.048790235	1.83857816	0.003000705	-1.948849814	1.50E-05	-3.787427974	3.92E-22
RP1	1.670174268	1.836568155	0.000216785	3.420441432	1.53E-16	1.583873277	5.44E-08
PSG1	4.277220214	1.835638797	0.006243357	6.125827762	1.40E-29	4.290188965	4.15E-25
IL22RA2	1.657618269	1.829666037	0.000226264	2.938224945	3.98E-13	1.108558908	0.000173388
FN1	17.52421496	1.821789771	0.000903223	7.001308772	2.73E-50	5.179519001	2.14E-44
GPR115	2.921955067	1.817033448	0.001930985	4.766074884	7.87E-26	2.949041436	9.15E-17
SAA1	2.308379612	1.812393727	0.002118269	3.058592496	7.94E-10	1.246198769	0.00057497
OMD	4.329646581	1.805940252	0.006329188	-1.631688668	0.00019943	-3.43762892	6.83E-18
VGLL1	3.862381928	1.803788099	0.003575182	5.47512369	5.98E-37	3.671335591	1.37E-21
CXCL5	2.839875677	1.796810477	0.001541752	4.494157253	2.57E-27	2.697346776	5.03E-15
SAA2	1.954986537	1.771771648	0.002550927	3.195101451	1.56E-10	1.423329803	4.95E-05
SLPI	10.02490438	1.741528304	0.002473199	4.303643324	5.15E-24	2.56211502	1.76E-13
ALDH3B2	2.694689126	1.717389343	4.67E-05	4.520543673	1.45E-30	2.80315433	4.99E-28
HLA-DQB2	8.071486394	1.699525179	0.000539641	4.020575243	7.02E-14	2.321050065	6.12E-13
COL1A1	13.25194687	1.693673844	0.00027576	3.621021585	4.18E-22	1.927347741	5.22E-12
CCL22	5.746767496	1.692568341	0.001424019	2.922097618	8.71E-10	1.229529277	0.000307643
FUT9	1.723128322	1.68621358	0.00561117	3.628259631	4.03E-14	1.942046051	2.84E-08

NMU	6.171298671	1.685383752	0.034740302	6.814441854	1.51E-25	5.129058103	6.03E-25
TNFSF18	1.725274972	1.684371423	0.000107785	3.618259255	1.09E-22	1.933887832	5.36E-14
SERPINB2	1.914597879	1.683057297	0.001222044	3.223836119	7.27E-15	1.540778822	6.08E-07
ADAM12	6.229578091	1.680029096	0.000199058	3.582736743	1.05E-15	1.902707646	3.15E-12
ADAMTS14	5.841033543	1.676155136	0.000814501	5.533319127	1.41E-27	3.85716399	4.32E-31
MMP11	7.912247583	1.654370095	6.42E-05	4.596746854	1.03E-29	2.942376759	8.64E-31
C15orf48	7.301892924	1.651775739	0.000216785	2.724845846	1.09E-15	1.073070108	5.68E-05
EREG	3.656928704	1.650006067	0.011523788	4.659977411	5.36E-19	3.009971344	3.05E-14
CD70	3.758236597	1.647803235	0.004589663	3.901359671	3.11E-14	2.253556436	5.83E-10
AHNAK2	10.62110081	1.642449775	6.42E-05	4.733698288	1.78E-44	3.091248513	1.90E-33
SDR16C5	3.436355887	1.622324639	0.007678955	4.335761616	3.08E-18	2.713436978	4.95E-13
SFN	8.329691586	1.605928821	0.004740177	5.244722847	2.14E-28	3.638794026	9.69E-24
KCNN4	9.315738395	1.602096027	0.003456311	5.645425749	3.07E-27	4.043329723	1.66E-28
KRT16	2.129724111	1.58424902	0.010717099	3.643049138	1.18E-12	2.058800117	8.84E-09
FUT3	4.144518766	1.581971862	0.003884889	5.696096181	2.54E-34	4.11412432	6.24E-32
11-Mar	1.347454188	1.572459949	0.000805208	2.897397281	7.72E-19	1.324937332	1.24E-06
SYT1	9.761840301	1.570011091	0.037070525	8.870015092	8.95E-43	7.300004002	3.75E-47
FIBIN	5.633609395	1.567050078	0.002792939	-1.264655695	8.00E-07	-2.831705772	1.35E-18
WDR69	1.390201967	1.540846604	0.000350494	2.975997998	3.18E-22	1.435151394	2.99E-08

LPPR1	1.286285363	1.538437335	0.000876923	3.159057121	2.26E-17	1.620619786	3.05E-09
KLK10	9.739873335	1.529589337	0.046804983	7.304834554	1.71E-36	5.775245217	2.40E-32
CAPN8	2.403985907	1.529389738	0.002506739	3.608721714	6.92E-18	2.079331976	2.92E-12
FAM101A	3.051134966	1.518351604	0.000356665	3.103624846	3.12E-18	1.585273242	1.00E-09
VCAN	9.078241797	1.516677498	0.000719447	2.708980737	4.37E-15	1.192303239	9.73E-06
CST5	3.148211113	1.505753038	0.007845872	4.766471157	1.77E-30	3.26071812	2.38E-21
COMP	8.454769032	1.497681186	0.038408231	5.761700321	5.30E-19	4.264019134	3.04E-21
CFTR	2.27216564	1.492025579	0.003035446	2.800355137	1.45E-18	1.308329558	1.62E-05
CTHRC1	7.421805003	1.488429072	0.001424019	2.882734515	9.80E-18	1.394305444	5.75E-07
CPNE4	5.123881869	1.486453347	0.036402619	5.455600093	2.70E-23	3.969146747	1.14E-19
SRPX2	5.293474362	1.476250187	0.000539641	2.75190293	1.22E-18	1.275652743	4.47E-07
C2CD4B	5.300427802	1.473941326	0.001955223	2.839320856	7.54E-11	1.36537953	4.60E-06
CDH22	5.608579738	1.466255776	0.007051992	4.02371571	1.68E-25	2.557459934	1.76E-14
CCR8	1.733775323	1.458817883	0.000653302	2.501091009	7.93E-12	1.042273126	9.05E-05
IGFL2	5.703059812	1.452328991	0.035921178	6.297405725	9.79E-25	4.845076735	9.39E-29
SPOCD1	5.003880699	1.436439884	0.000857659	4.173691429	2.42E-33	2.737251545	5.85E-24
DCN	10.1306421	1.435944826	0.006676868	-1.47642911	1.22E-06	-2.912373936	2.38E-19
OR13A1	1.674188488	1.422586914	0.000710045	2.963419056	1.13E-14	1.540832142	3.13E-09
MUC1	10.80998662	1.409017621	0.001572993	3.414701895	2.03E-27	2.005684275	2.93E-13

ASPHD1	5.033155077	1.406519	0.001899081	5.472719016	3.84E-34	4.066200015	5.47E-40
PHACTR3	4.014218161	1.392446254	0.008845824	4.23875943	8.87E-26	2.846313175	6.15E-18
MATN3	4.501425569	1.389630667	0.000199058	2.626809997	9.58E-20	1.237179331	1.01E-08
PLAU	11.33822923	1.387227459	0.000505741	4.3316127	1.22E-35	2.944385241	1.86E-30
LRRN4	1.943658793	1.386500623	0.001740515	2.420419325	4.71E-13	1.033918702	0.000123659
S100A2	8.4240234	1.373060538	0.000439835	2.631148873	3.54E-14	1.258088335	2.55E-08
CLDN16	8.570253971	1.367041823	0.031489519	6.535760065	6.99E-36	5.168718242	4.27E-36
GALNT5	4.54593732	1.36618653	0.010128103	4.126991761	1.54E-25	2.760805231	4.81E-17
C19orf21	6.762978586	1.365263774	0.010661052	4.749943583	7.87E-26	3.38467981	7.85E-23
ADRB3	1.137277511	1.361119646	0.000332484	2.633596619	6.23E-21	1.272476973	2.02E-08
NKD2	4.038464453	1.355612009	0.002118269	2.931688019	1.34E-12	1.57607601	4.83E-09
GOLGA7B	7.279480294	1.355250952	0.000331801	3.392516895	5.23E-21	2.037265942	5.88E-17
TMEM100	7.283089624	1.354651279	0.0063401	3.358905639	3.53E-24	2.004254359	2.93E-11
P2RY6	6.750494429	1.353023434	8.75E-05	3.076702774	5.54E-22	1.723679339	1.16E-15
BIRC7	5.197805479	1.338475448	0.044017681	5.876282236	4.68E-27	4.537806788	3.39E-28
COL5A1	9.7638314	1.329175504	0.000857659	2.466110303	1.73E-15	1.136934799	1.32E-06
SERPINA1	14.83397139	1.321066158	0.004288164	5.714351026	2.14E-46	4.393284869	1.76E-44
ABCA12	1.929960235	1.315670894	0.002689485	3.606912905	2.47E-23	2.291242011	3.63E-18
MARCO	6.974142366	1.312170455	0.033996435	3.950471129	2.95E-11	2.638300674	1.57E-11

ALOX15B	9.36686668	1.311910138	0.011290811	4.353356919	1.94E-23	3.041446781	1.74E-20
DEPDC1B	6.968400741	1.311707911	0.00251955	2.949248017	3.45E-18	1.637540106	1.49E-09
SFTA2	4.079811946	1.310618119	0.007144775	3.482863197	2.89E-15	2.172245077	5.19E-13
HES2	4.689424186	1.30716547	0.002915458	4.814458181	7.90E-25	3.50729271	7.90E-33
C11orf41	3.960257573	1.301080303	0.004157766	2.58927207	1.17E-15	1.288191767	2.22E-06
PLAC2	4.684879325	1.300109708	0.030096391	4.71720214	7.21E-23	3.417092432	1.89E-21
TNFSF11	3.266628391	1.294811646	0.014596074	3.027217206	1.01E-08	1.73240556	4.42E-07
STAC	6.495710867	1.291920575	0.005123022	4.291421837	6.76E-34	2.999501262	7.88E-25
COL19A1	3.17150888	1.291564574	0.027386031	3.439255297	5.12E-10	2.147690723	2.08E-09
CYP2S1	8.042059853	1.29138732	0.006022103	4.859123248	1.48E-33	3.567735927	7.24E-31
GPR87	2.433320537	1.281276884	0.010521459	3.729595342	1.02E-16	2.448318458	7.80E-17
IL1F5	4.261174232	1.2798147	0.002901	4.975657396	7.40E-34	3.695842696	4.72E-38
PNP	10.5061761	1.278440388	7.07E-05	2.889194022	2.50E-21	1.610753634	3.13E-17
TRIM63	3.906260547	1.272894957	0.010468451	4.287356843	9.47E-18	3.014461886	5.32E-21
DHDH	4.074734443	1.267213901	0.002984667	4.614342957	8.21E-30	3.347129056	6.40E-33
SLC18A2	1.214780257	1.266334258	0.003533398	2.700398873	7.30E-14	1.434064615	2.14E-08
CRLF2	2.554345384	1.266102971	0.018116141	3.842269448	6.16E-26	2.576166477	4.31E-16
CCL13	5.680330974	1.259882722	0.018399612	4.025941243	1.97E-20	2.766058521	1.75E-16
SEZ6L2	8.909687294	1.255991303	0.003157621	4.7194018	3.42E-38	3.463410497	3.14E-35

CCNA1	4.862400424	1.253615881	0.011582582	3.836589826	1.55E-22	2.582973944	2.17E-17
ACTBL2	3.953926924	1.244544592	0.010073206	5.358417279	5.14E-35	4.113872688	5.05E-39
TACSTD2	12.46610108	1.242449078	0.032384004	5.25003778	3.22E-34	4.007588703	1.09E-27
AZU1	1.226414866	1.235042741	0.013253846	2.755714839	1.32E-13	1.520672098	1.28E-07
SPRR1B	1.371508124	1.234167222	0.024073571	2.675020083	3.59E-11	1.440852862	3.65E-06
CSF2	3.847096644	1.229926183	0.008618867	5.116170296	1.55E-40	3.886244113	4.50E-38
DCHS2	4.879281342	1.22552968	0.00101595	3.34341428	1.58E-24	2.1178846	1.23E-19
EBI3	6.162932071	1.212497911	0.000821291	3.429037945	3.85E-14	2.216540034	5.23E-19
F2RL2	3.971469873	1.204220549	0.00906528	3.19083869	1.42E-13	1.98661814	1.46E-12
RHOV	5.914172246	1.195506254	0.001810068	2.899975181	1.54E-22	1.704468927	3.38E-13
IL1RN	7.982234381	1.192284055	0.001756707	2.530390202	4.48E-18	1.338106147	1.14E-08
C10orf55	5.271741593	1.19007139	0.002047094	4.156376447	7.86E-34	2.966305058	4.96E-32
FLJ42709	7.883465526	1.187332674	0.001126081	3.765851064	4.53E-48	2.57851839	1.33E-28
DUSP13	3.221052921	1.183409009	0.019506265	4.472676914	1.77E-27	3.289267905	1.04E-25
PVRL4	8.986144325	1.182082718	0.006185661	5.961319082	6.12E-42	4.779236364	1.16E-54
CNTN1	1.425833312	1.181956728	0.024427667	-2.327380738	8.49E-08	-3.509337466	1.24E-27
SOX11	3.760598523	1.181063044	0.025430755	4.306345272	8.30E-23	3.125282227	2.23E-21
HLA-G	8.068078796	1.172544464	0.012528858	2.974899018	1.01E-13	1.802354554	4.67E-10
CFB	10.8327853	1.171573206	0.020986707	2.653477997	1.86E-13	1.481904791	2.09E-06

ARSE	2.759092984	1.168439436	0.001896895	2.877834432	1.99E-17	1.709394995	7.92E-14
CDH4	5.634307555	1.164075429	0.038517644	4.594317228	1.63E-23	3.430241799	1.16E-22
C19orf33	9.358542382	1.162315207	0.005238996	3.458032382	7.56E-24	2.295717175	1.74E-18
CITED1	11.33963777	1.160806098	0.036747747	5.791456585	1.19E-30	4.630650486	6.96E-39
EPHA10	7.191945435	1.156174766	0.008550866	5.265966267	9.56E-42	4.109791502	6.89E-44
KRT19	12.37926227	1.150001118	0.004740177	3.507209002	1.01E-32	2.357207884	1.83E-20
NGEF	9.320597238	1.140681914	0.019287413	6.577108548	3.64E-42	5.436426634	5.54E-57
CDKN2A	5.736289159	1.139910676	0.004432694	4.190978475	3.39E-21	3.051067799	2.12E-31
GNLY	7.18175996	1.138048196	0.018026444	2.7901058	1.10E-10	1.652057605	1.35E-08
SULT2B1	6.877081228	1.13803458	0.003349664	2.821010891	2.62E-17	1.682976311	2.71E-12
CDKN2BAS	1.643407151	1.136439603	0.001202483	2.713726625	2.12E-22	1.577287022	4.44E-13
HCN4	6.246659077	1.132096441	0.009410435	5.955758728	6.99E-36	4.823662287	3.32E-54
TNC	10.83286418	1.125292004	0.009236398	3.412659945	7.82E-17	2.287367941	1.91E-16
TNFAIP6	4.424288193	1.12342333	0.002648959	3.219803519	4.98E-13	2.096380188	5.69E-18
UNC5CL	7.054706499	1.117337586	0.000541541	3.695403073	1.12E-38	2.578065487	2.03E-34
WBSCR26	4.984806455	1.113067225	0.011569754	3.103155232	1.36E-16	1.990088008	4.89E-13
CD164L2	5.696555244	1.112972898	0.018514791	4.844109595	5.35E-27	3.731136696	3.26E-34
LAMB3	11.41986097	1.111770288	0.01986355	5.452836589	3.78E-38	4.341066301	2.54E-42
SPTBN2	8.355354356	1.107203484	0.001496801	4.989481855	2.26E-40	3.882278372	5.80E-55

LPPR3	1.503731092	1.104657186	0.00744341	2.906033522	2.40E-20	1.801376336	2.62E-13
FAM178B	4.94821449	1.082272837	0.021238967	6.000008102	7.94E-43	4.917735265	7.71E-53
FBLN1	9.13713764	1.081902675	0.015433376	-1.521532467	8.68E-06	-2.603435141	7.53E-22
HPGDS	5.091518017	1.080066389	0.000625107	2.749365198	5.99E-20	1.669298808	9.45E-17
OR4D10	2.199452884	1.075360887	0.020089447	3.25125239	1.24E-23	2.175891503	1.03E-15
AQP9	4.612849666	1.075131246	0.013279782	2.579876153	1.03E-09	1.504744907	4.42E-08
VSTM2L	7.641089644	1.068865697	0.006223388	3.300944014	3.35E-23	2.232078317	5.23E-20
EN1	1.365641888	1.067328602	0.004746329	2.317988862	3.52E-13	1.25066026	5.80E-08
FAT2	5.761954191	1.06479009	0.006744918	2.224959656	8.80E-12	1.160169566	8.35E-07
BEAN	6.908997928	1.062601274	0.001766101	3.310918341	4.12E-34	2.248317067	2.12E-25
PMAIP1	6.830916855	1.046533287	0.002524124	2.70618231	6.11E-14	1.659649024	1.92E-13
MRAP2	1.11634961	1.046216921	0.00517113	-1.03993126	0.000390357	-2.086148181	7.99E-20
PPEF1	2.560441459	1.029534677	0.007444646	2.47249486	1.94E-15	1.442960183	3.73E-10
PDLIM4	10.96114957	1.026715927	0.023335593	4.374537311	2.37E-40	3.347821384	4.14E-31
SPINK2	4.427217697	1.025636932	0.004699801	3.342294673	4.14E-15	2.316657741	3.58E-22
HLA-DOB	6.157465548	1.025626396	0.018378845	2.15767322	0.000116174	1.132046824	0.000189192
GABRB2	10.46589561	1.021196142	0.043081875	8.501323154	2.90E-48	7.480127012	2.69E-83
SLC30A2	9.765677441	1.01951476	0.013387784	2.168949435	6.41E-14	1.149434675	5.01E-06
IBSP	1.719116326	1.009092797	0.033778904	2.868768226	1.32E-15	1.859675429	6.74E-12

AIM1L	3.859741793	1.003154926	0.010318736	3.62686279	1.89E-21	2.623707864	1.43E-26
MPPED2	6.193477822	-1.015150415	0.037141457	-4.982113739	9.11E-39	-3.966963324	3.96E-38
KIT	7.844291224	-1.016096753	0.018388435	-4.410472539	2.29E-26	-3.394375786	1.06E-36
HLF	7.660476647	-1.017502893	0.006185661	-3.504327475	1.42E-29	-2.486824582	3.34E-27
ATP4A	1.47313169	-1.02109402	0.016390792	-3.288968587	3.28E-22	-2.267874566	3.60E-18
C5orf23	6.939636263	-1.024402815	0.020810353	-2.91341443	1.05E-19	-1.889011615	1.59E-12
ZSCAN1	3.890828492	-1.024614933	0.000649169	-2.21918922	9.00E-20	-1.194574287	4.99E-11
LOC28479	0.710698247	-1.029134808	0.003849029	-2.878428297	2.30E-25	-1.849293489	4.93E-17
RYR2	5.053383747	-1.029669854	0.036124693	-5.728016197	4.18E-35	-4.698346342	7.85E-49
TRPM5	1.98861564	-1.029731937	0.010560213	-3.352338302	2.32E-17	-2.322606365	6.66E-21
UTS2R	2.541732638	-1.029820073	0.041638922	-3.03200632	4.53E-09	-2.002186247	3.89E-10
COLEC11	5.215060343	-1.030814417	0.00870923	-2.431155083	8.04E-16	-1.400340665	1.62E-09
CCDC108	3.656591644	-1.03128571	0.007267941	-2.285444139	7.27E-16	-1.254158428	4.89E-08
WBSCR17	7.143694848	-1.032369298	0.029296639	-2.692975875	1.18E-19	-1.660606576	2.35E-09
BTBD11	8.29107075	-1.035346225	0.000964924	-2.997280493	3.47E-34	-1.961934268	5.00E-24
TDH	1.332289149	-1.035894229	0.017594595	-4.032472958	1.82E-30	-2.996578729	5.63E-28
KATNAL2	6.060974004	-1.037312909	0.000226264	-3.079004882	6.17E-33	-2.041691973	7.76E-31
RSPH6A	5.299063825	-1.043632996	0.010407824	-2.44100666	2.25E-17	-1.397373663	5.25E-09
LRRC7	3.706055993	-1.056286551	0.032782245	-4.035533724	4.12E-23	-2.979247173	5.31E-24

LOC10014	1.606945666	-1.058590371	0.00282432	-2.371710255	2.50E-16	-1.313119885	1.67E-09
GABRG1	1.570337992	-1.063836508	0.02322313	-2.176089968	5.57E-11	-1.11225346	8.89E-05
C10orf82	4.077279236	-1.064692526	0.025608787	-2.678885265	2.08E-13	-1.614192739	4.10E-09
MYT1	4.167088331	-1.066442942	0.001208106	-2.322652507	1.67E-17	-1.256209565	2.17E-10
SRRM4	1.039842673	-1.073044215	0.030382843	-2.667839375	3.14E-16	-1.59479516	1.15E-07
SFTPC	1.395089866	-1.07619473	0.040627807	-4.543990841	5.20E-27	-3.467796111	5.51E-27
90288	4.517773756	-1.083828933	7.15E-05	-2.138765972	1.07E-18	-1.054937039	3.09E-11
C1QTNF4	1.616098784	-1.084283867	0.013390073	-2.892917343	3.15E-16	-1.808633476	6.44E-12
FLJ42875	5.608293008	-1.086389698	0.001565276	-3.207103986	1.62E-29	-2.120714289	3.54E-23
NEB	4.602429912	-1.08780668	0.041586707	-4.532542199	4.05E-23	-3.444735519	2.66E-24
CECR2	2.449726868	-1.091116325	0.012943683	-4.481108776	6.08E-25	-3.389992451	1.22E-33
KIF19	3.054740082	-1.093560197	0.04716767	-4.964175151	2.05E-29	-3.870614954	1.55E-30
CLCNKA	4.398790804	-1.109265728	0.029395296	-4.542022425	7.76E-33	-3.432756697	1.01E-27
DIRAS2	3.864845778	-1.118279803	0.006744918	-4.438126104	1.06E-34	-3.319846302	3.57E-36
FHL1	10.96614835	-1.122195903	0.00177173	-3.800572504	2.85E-37	-2.678376601	4.37E-32
SERHL2	3.6823272	-1.122313469	0.001304987	-3.724715746	9.24E-22	-2.602402277	2.36E-30
LRRC50	3.510254364	-1.123878352	0.00136219	-4.003356614	5.75E-32	-2.879478263	5.08E-37
AGXT2L1	0.713960122	-1.127231893	0.026874255	-4.860594499	3.32E-31	-3.733362606	3.08E-30
CXXC4	2.341035509	-1.13597438	0.002251409	-3.083628046	7.34E-17	-1.947653666	4.11E-17

NOL4	1.832107608	-1.143730667	0.041288678	-5.663419142	3.65E-31	-4.519688475	3.22E-37
SLC38A3	4.900662325	-1.154655887	0.024192627	-2.274391634	2.63E-13	-1.119735747	0.000187533
C20orf56	0.865689846	-1.158982204	0.008163204	-2.838176353	2.67E-11	-1.679194149	7.92E-10
BEX1	6.080195157	-1.161193735	0.020836028	-4.169031458	1.67E-29	-3.007837723	1.86E-22
PYGM	5.032228825	-1.161783481	0.016642916	-3.597391216	3.16E-26	-2.435607735	8.19E-17
SPINK5	3.365843266	-1.166400842	0.037544193	-2.797866314	2.72E-07	-1.631465472	1.58E-06
CD36	8.004539076	-1.169382079	0.0050683	-2.942390417	3.12E-20	-1.773008339	5.81E-13
LOC25455	4.260411325	-1.170750526	0.04947947	-3.248517836	9.40E-15	-2.077767309	1.74E-09
GABRB3	6.195232278	-1.182529946	0.02756969	-3.934110581	4.43E-21	-2.751580635	3.57E-18
LIX1	5.775731246	-1.193047693	0.047103343	-3.032663236	4.91E-14	-1.839615542	2.50E-07
IRS4	1.690561218	-1.210206838	0.011020387	-4.987679982	1.96E-24	-3.777473145	2.94E-33
GCK	2.006159134	-1.211285026	0.004710521	-3.174374273	3.72E-20	-1.963089247	1.39E-13
APOA1	3.073454245	-1.233598848	0.020894416	-3.981016489	1.30E-23	-2.74741764	1.46E-17
KIF5A	3.299530772	-1.234236283	0.037806784	-2.613243104	5.29E-08	-1.37900682	0.000136897
SLC14A2	2.359123287	-1.237594265	0.0347176	-2.916568293	1.68E-13	-1.678974028	2.09E-06
GPR158	2.614082395	-1.244760515	0.003604745	-2.76009704	3.29E-16	-1.515336525	9.19E-09
COL9A3	5.859605618	-1.269582644	0.045168558	-4.808392525	7.70E-18	-3.538809881	1.36E-19
FAM167A	8.820425469	-1.2724462	0.004740177	-4.342740254	1.32E-28	-3.070294054	2.36E-27
SLC26A7	10.44461739	-1.275440041	0.016595618	-5.048388392	5.13E-25	-3.772948351	7.87E-31

FOXA2	4.243292214	-1.276712194	0.046868551	-4.798704267	5.01E-23	-3.521992074	4.76E-20
NPR3	4.776103547	-1.277029122	0.011221724	-3.505337316	6.58E-18	-2.228308194	4.97E-13
KHDRBS2	3.843867088	-1.278772407	0.006587165	-4.728510181	9.61E-30	-3.449737774	2.62E-31
UPF0639	1.371822085	-1.292674331	0.011589552	-5.211647155	7.50E-36	-3.918972825	4.13E-33
NWD1	2.969101808	-1.294183643	0.028087588	-5.636627112	6.30E-31	-4.342443468	1.40E-32
C12orf39	2.149780156	-1.297944624	0.011569754	-5.265916438	1.63E-32	-3.967971814	9.21E-34
UNC5D	2.231888277	-1.29820735	0.011034859	-2.704646695	2.77E-10	-1.406439345	1.12E-05
TPTE2P1	3.461706131	-1.303972695	0.013718542	-5.056772406	3.94E-29	-3.752799712	2.75E-29
C18orf16	1.706346578	-1.319451075	0.002931882	-2.46851107	4.36E-17	-1.149059995	1.91E-05
DNASE1L3	6.021095295	-1.325794007	0.000895175	-2.615477365	3.24E-15	-1.289683358	5.30E-08
ZNF560	2.332410033	-1.345890806	0.030825776	1.389351504	6.25E-05	2.735242311	7.24E-14
DKFZP434	2.115707093	-1.354370361	0.00185329	-2.469594885	1.12E-13	-1.115224525	2.25E-05
SLC4A4	7.380104284	-1.357440299	0.00337746	-5.174470384	2.06E-33	-3.817030085	2.53E-38
LRRTM1	2.959084575	-1.362404187	0.03779708	-3.333726857	4.80E-12	-1.971322669	5.78E-07
SELV	2.146775613	-1.363614849	0.024732188	-5.545031903	4.81E-36	-4.181417053	8.06E-29
TRPM3	0.855939207	-1.367677688	0.00243958	-5.089146853	1.55E-32	-3.721469165	4.51E-36
ZNF536	1.814294611	-1.370929796	0.016569151	-5.644863916	2.35E-37	-4.27393412	1.35E-32
LOC10013	3.459103307	-1.402050748	0.007507122	-4.918367111	5.37E-27	-3.516316363	5.93E-28
MRO	3.791583503	-1.414434994	0.013673064	-6.228919	4.76E-39	-4.814484006	1.67E-39

CIB4	0.914116274	-1.456136209	0.001346485	-4.076755508	3.97E-31	-2.6206193	6.30E-20
LOC28600	9.897232779	-1.49669228	0.026059007	-5.101336319	7.71E-26	-3.604644039	1.39E-19
C7orf52	2.616636046	-1.516144301	0.01134709	-4.235785879	1.90E-20	-2.719641577	4.76E-14
ABCC8	3.750668059	-1.517554215	0.03434172	-4.582507045	2.61E-20	-3.064952829	6.94E-13
HS6ST3	4.175316134	-1.540194976	0.00185329	-4.978811765	5.92E-32	-3.438616789	2.44E-28
STXBP5L	3.960315259	-1.556183753	0.013387784	-6.495549344	2.11E-34	-4.939365591	1.24E-35
PKNOX2	5.683304527	-1.571015621	0.026593714	-5.326950746	2.04E-25	-3.755935126	3.23E-19
CHRNA4	2.877808302	-1.597142164	0.01201356	-5.050288841	1.50E-22	-3.453146677	3.14E-19
KCNJ13	3.560246396	-1.5993816	0.004904046	-4.604081719	1.05E-21	-3.004700119	2.03E-17
KCNAB1	7.919545218	-1.608148874	0.000541541	-3.786675765	3.52E-29	-2.178526891	1.07E-14
GRIK4	3.557094074	-1.626204335	0.001346485	-4.894848464	9.61E-30	-3.26864413	1.37E-24
WNT4	4.684610788	-1.659186576	0.000361761	-3.820439432	2.13E-22	-2.161252856	3.97E-14
TRPV6	4.027897744	-1.691575485	0.000454108	-3.098558314	1.68E-23	-1.406982829	1.13E-06
FLRT1	4.168981918	-1.717436169	0.000568042	-5.29531421	2.19E-33	-3.577878041	8.62E-30
LECT1	3.252696424	-1.752827084	0.020575083	-4.767826941	4.26E-21	-3.014999856	1.96E-11
FOXJ1	3.714437	-1.76983176	0.004163456	-5.838354277	1.77E-27	-4.068522518	9.70E-26
PKHD1L1	6.173578035	-1.88419602	0.032193638	-7.876580457	4.62E-29	-5.992384437	4.57E-29
LOC10013	3.030855369	-1.892567595	0.000294649	-2.933111897	6.10E-15	-1.040544302	0.000908078
TPO	11.12584274	-1.975085722	0.005288505	-6.925495438	4.59E-32	-4.950409716	8.93E-30

EDN3	5.396335421	-2.334551611	0.004041661	-6.200850521	2.34E-23	-3.866298911	1.87E-15
CA4	4.957288696	-2.648212423	0.000332484	-6.191657395	3.13E-26	-3.543444972	1.01E-15

Supplementary Table S3. Enriched GO and KEGG terms of 25 genes identified in the three subnetworks and 8 hub genes.

Pathway ID	pathway description	observed gene count	false discovery rate
**For 25 genes in the subnetworks**			
**GO: Cell component**			
GO.000561	extracellular space	16	3.34E-11
GO.000558	collagen trimer	8	5.83E-11
GO.009864	complex of collagen trimers	5	2.52E-08
GO.000557	proteinaceous extracellular matrix	9	3.65E-08
GO.000558	fibrillar collagen trimer	4	1.99E-07
GO.009864	banded collagen fibril	4	1.99E-07
GO.004442	extracellular region part	18	3.68E-07
GO.000557	extracellular region	18	5.51E-06
GO.000578	endoplasmic reticulum lumen	6	1.44E-05
GO.004442	extracellular matrix component	5	3.88E-05
**GO: Biological process**			
GO.003019	extracellular matrix organization	13	1.90E-13
GO.002261	extracellular matrix disassembly	9	2.78E-11
GO.003057	collagen catabolic process	8	4.43E-11
GO.000960	response to external stimulus	15	1.40E-07
GO.002241	cellular component disassembly	10	1.64E-07
GO.000693	chemotaxis	10	9.08E-07
GO.004471	single-organism catabolic process	10	1.90E-05
GO.006032	cell chemotaxis	6	2.87E-05
GO.001647	cell migration	9	6.85E-05
GO.005167	localization of cell	9	0.000128
GO.004001	locomotion	10	0.000215
GO.000150	ossification	6	0.000268
GO.000695	immune response	10	0.000476
GO.000692	movement of cell or subcellular component	10	0.000482

GO.004222	response to chemical	15	0.000633
GO.000961	response to mechanical stimulus	5	0.0018
GO.006144	connective tissue development	5	0.002
GO.006035	cartilage development involved in endochondral bone morphogenesis	3	0.00227
GO.000726	cell-cell signaling	8	0.00274
GO.005170	response to other organism	7	0.00359
GO.000961	response to bacterium	6	0.00483
GO.003019	collagen fibril organization	3	0.0057
GO.007088	cellular response to chemical stimulus	11	0.0057
GO.003249	response to lipopolysaccharide	5	0.00597
GO.003399	response to lipid	7	0.00766
GO.003296	collagen biosynthetic process	2	0.00821
GO.000237	immune system process	10	0.00955
GO.000988	tissue development	9	0.00955
GO.003598	tendon development	2	0.0106
GO.005121	cartilage development	4	0.0107
GO.006035	endochondral bone morphogenesis	3	0.016
GO.000715	cell adhesion	7	0.0225
GO.000905	catabolic process	9	0.0225
GO.003121	biomineral tissue development	3	0.0289
GO.004860	reproductive structure development	5	0.0289
GO.000695	response to stress	12	0.0294
GO.006145	reproductive system development	5	0.0294
GO.000695	inflammatory response	5	0.0302
GO.001604	cellular component organization	14	0.0302
GO.004470	single organism reproductive process	7	0.0323
GO.001003	response to organic substance	10	0.0424
GO.000341	growth plate cartilage development	2	0.0435
**GO: Molecular function**			
GO.000520	extracellular matrix structural constituent	6	5.13E-07
GO.000800	chemokine activity	5	3.42E-06
GO.000512	cytokine receptor binding	7	3.89E-06

GO.000512	cytokine activity	6	1.62E-05
GO.000551	protein binding	17	0.000352
GO.000553	glycosaminoglycan binding	5	0.00154
GO.000510	receptor binding	8	0.00975
GO.000820	heparin binding	4	0.0109
GO.004840	platelet-derived growth factor binding	2	0.0269
GO.004339	proteoglycan binding	2	0.05
**KEGG pathways**			
4060	Cytokine-cytokine receptor interaction	8	1.03E-07
4512	ECM-receptor interaction	6	1.03E-07
4062	Chemokine signaling pathway	7	1.35E-07
4510	Focal adhesion	6	9.35E-06
4151	PI3K-Akt signaling pathway	6	0.000135
4974	Protein digestion and absorption	4	0.000135
5323	Rheumatoid arthritis	4	0.000135
5146	Amoebiasis	4	0.000263
4611	Platelet activation	4	0.000501
4064	NF-kappa B signaling pathway	3	0.00474
4668	TNF signaling pathway	3	0.00747
5206	MicroRNAs in cancer	3	0.0164
**For 8 hub genes**			
**GO: Cell component**			
GO:0005581	collagen trimer	5	1.06E-07
GO:0005788	endoplasmic reticulum lumen	5	2.86E-06
GO:0005583	fibrillar collagen trimer	3	3.53E-06
GO:0098643	banded collagen fibril	3	3.53E-06
GO:0005578	proteinaceous extracellular matrix	4	0.00111
GO:0005783	endoplasmic reticulum	5	0.0159
**GO: Biological process**			
GO:0030574	collagen catabolic process	5	1.93E-07
GO:0022617	extracellular matrix disassembly	5	5.71E-07
GO:0030199	collagen fibril organization	3	0.000504
GO:0040011	locomotion	6	0.000726

GO:0006935	chemotaxis	5	0.00107
GO:0032964	collagen biosynthetic process	2	0.00198
GO:0035989	tendon development	2	0.00258
GO:0009605	response to external stimulus	6	0.00656
GO:0006928	movement of cell or subcellular component	5	0.0266
GO:0061448	connective tissue development	3	0.034
**GO: Molecular function**			
GO:0005201	extracellular matrix structural constituent	4	2.48E-05
GO:0048407	platelet-derived growth factor binding	2	0.0151
**KEGG pathways**			
4974	Protein digestion and absorption	4	5.25E-06
4512	ECM-receptor interaction	3	0.000548
5146	Amoebiasis	3	0.000671
4611	Platelet activation	3	0.000896
4062	Chemokine signaling pathway	3	0.00202
4510	Focal adhesion	3	0.00245
4060	Cytokine-cytokine receptor interaction	3	0.00444
4151	PI3K-Akt signaling pathway	3	0.0083
5323	Rheumatoid arthritis	2	0.015
4668	TNF signaling pathway	2	0.0209
